# Electrohydraulic Folding Ring Actuators for Radially Contracting Applications

**DOI:** 10.1002/advs.75613

**Published:** 2026-05-11

**Authors:** Gavril Yong En Tan, Quan Xiong, Jonathan William Ambrose, Zeynep Altundal, Raye Chen‐Hua Yeow

**Affiliations:** ^1^ Department of Biomedical Engineering National University of Singapore Singapore Singapore; ^2^ Department of Electromechanical Engineering (EME) and Centre for Artificial Intelligence and Robotics (CAIR) Faculty of Science and Technology University of Macau Macau SAR China; ^3^ Department of Mechatronics Engineering Faculty of Engineering and Natural Sciences Sabancı University Istanbul Turkey

**Keywords:** bending actuators, constricting actuators, electrohydraulic actuators, folding actuators, radially contracting actuators

## Abstract

Soft radially contracting actuators, inspired by biological circular muscles, enable diverse robotic functions, including manipulation, gut simulation, locomotion, and haptics. The combination of electrohydraulic actuation with radially contracting actuators holds promise for the development of high‐performance radial contraction in a compact form factor. In this paper, we introduce an electrohydraulic folding ring actuator (EFRA) that utilizes folding motion to actively close its inner lumen. The EFRA is lightweight (∼25.9 g) and compact, consisting of a triangular ring with sides of ∼65 mm. Under 7 kV actuation, the EFRA achieves a high contraction ratio of 0.89 sustained over 5 s of actuation. This performance is enabled by the multiple synchronously bending segments in the folding mechanism, which enhances radial contraction compared with prior electrohydraulic radially contracting actuator designs. The EFRA also produces ∼0.96 N of inward‐directed force measured at a single inner vertex under 7 kV actuation, yielding high force output relative to its low mass. Finally, we demonstrate the advantages of the high performance and compact nature of the EFRA in multiple applications, including robotic manipulation, locomotion, as well as an artificial robotic sphincter.

## Graphical abstract

This work presents an Electrohydraulic Folding Ring Actuator that combines high‐performance electrohydraulic actuation with origami‐inspired folding geometry to achieve constricting radial actuation. This integration yields significant inner lumen constriction, alongside a gripping force capable of holding objects exceeding four times the actuator's mass while maintaining a compact form factor. This architecture enables robotic applications including manipulation, locomotion, and artificial sphincters.

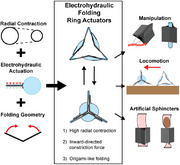

## 1 Introduction

Radial contracting soft actuators are often inspired by the function of biological circular muscles [1, 2, 3, 4, 5], which in nature serve as a constricting mechanism for a variety of different functions. This ranges from sphincters and peristaltic transport in the gut [6, 7, 8], to locomotion such as peristaltic crawling in earthworms [9], or jet propulsion swimming in cephalopods [10], and jellyfish [11]. Similarly to their biological counterparts, soft radially contracting actuators (RCAs) are ring‐shaped or tube‐shaped actuators used primarily for their inward constriction of an inner lumen, which can be used for robotic manipulation [2, 12, 13, 14, 15, 16, 17], gut simulation [4, 5, 18, 19, 20, 21, 22], sphincter models [1, 23, 24], fluidic pressure generation [25, 26, 27], and heart assistance [28, 29], robotic locomotion [2, 3, 30, 31, 32], or even as haptic devices [33].

Despite the range of applications that radially contracting soft actuators may be applied to, they are a relatively less researched field compared to more common soft actuator deformation types such as bending actuators, or muscle‐unit inspired longitudinally contracting actuators. Nevertheless, there have still been many attempts to develop radially constricting actuators using a variety of different soft actuation technologies, such as pneumatics [12, 13, 18], shape memory alloys [3], or even electrostatic‐based actuation [2, 27]. Beyond soft actuators, attempts have also previously been made to recreate the constricting motion using rigid robotic components [34].

While many of these actuators are able to achieve high radial contraction performance or high inward‐directed force output which is essential for applications such as artificial sphincters or robotic manipulation, they can be bulky in their construction. Pneumatic soft actuators are one of the most used actuator types used to make radially contracting actuators. However, they often require rigid pumps [35] or rigid strain‐limiting exteriors [1, 14, 24], with other McKibben‐based designs exhibiting high forces but limited inward contraction [12, 28]. Shape memory alloys (SMAs) such as Nitinol may offer a lighter alternative but can suffer from slow actuation speeds due to being thermally controlled [36, 37], which can also limit their application. Furthermore, many radially contracting actuators are also limited to single specific applications due to their bulky design, limited inward contraction, or application‐specific design, with only a few examples showcasing multiple applications [2, 32]. Accordingly, there remains a lack of radially contracting soft actuators that achieve large lumen closure with fast response, while retaining a compact form factor that facilitates integration into different robotic applications.

Alternatively, radially contracting soft actuators that utilize electrostatic‐based actuation offer potential for actuators with high strain and force capabilities, directly controlled by electrical signals with fast response time [38, 39] that can allow them to have a wider range of potential applications. Dielectric elastomer actuators (DEAs) for instance have been used to develop lightweight radially contracting ring‐like actuators using bidirectional bending DEA segments, with sufficient radial contraction and actuation speed, for a variety of applications such as locomotion as well as manipulation with a high payload‐to‐weight ratio of 5.3 [2]. However, it relies on passive elastic closure of the ring structure, limiting performance such as low maximum manipulation payloads.

Electrohydraulic actuation is another electrostatic‐based actuation technology that shows promise in developing radially contracting soft actuators. Electrohydraulic actuators, also known as hydraulically amplified self‐healing electrostatic (HASEL) actuators, have gained much recent attention due to its high performance in a very compact form factor [38, 40], with the potential to develop a variety of actuator geometries through both linearly contracting [27, 41], or bending deformation [42]. HASEL‐based radially contracting actuators bring potential for the development of highly compact, fast response, and high‐performance actuators for constriction tasks. Such actuators eliminate the need for bulky external components such as pumps and compressors as in the case of pneumatic radially contracting actuators [35], allow for high‐speed actuation due to being electrically powered [41] in contrast with other actuation methods such as SMAs or pneumatic actuators, while retaining a relatively compact form factor.

Linear contracting geometries typically utilize Peano‐HASEL type actuators that, when a high voltage is applied to the electrodes, cause expansion lengthwise contraction of the actuating portion of the electrohydraulic pouch [41]. Prior attempts have been made to develop radially contracting actuators using the Peano‐HASEL geometry [23, 27] due to its mechanical similarity to lengthwise contraction of biological muscles. However, they exhibit limited inward radial contraction, with one achieving a maximum theoretical contraction ratio of 0.59 [27] and the other also exhibiting limited but unmeasured inward radial contraction [23], limiting their range of application.

Instead of using linearly contracting elements to achieve radial contraction, a series of bending actuators is also capable of achieving closure of an inner lumen. Bidirectional bending DEAs arranged in a ring were previously used to form a contracting ring structure, although this relied on elastic passive closure of the ring [2]. Pneumatic bending actuators were also used to form a closing ring with active inward force output [16], but still visually exhibited large inner lumen gaps during maximum closure, and had a relatively large and bulky design alongside requiring the associated heavy compressors and valves.

An alternative method is the use of bending HASEL‐actuator geometries, first known as spider‐inspired electrohydraulic soft‐actuated (SES) joints [42]. Such actuators maintain the benefits of HASEL actuators such as high specific power, light weight, and fast response, while also having high rotation angles which may be leveraged for other high‐strain designs [43].

**FIGURE 1:**
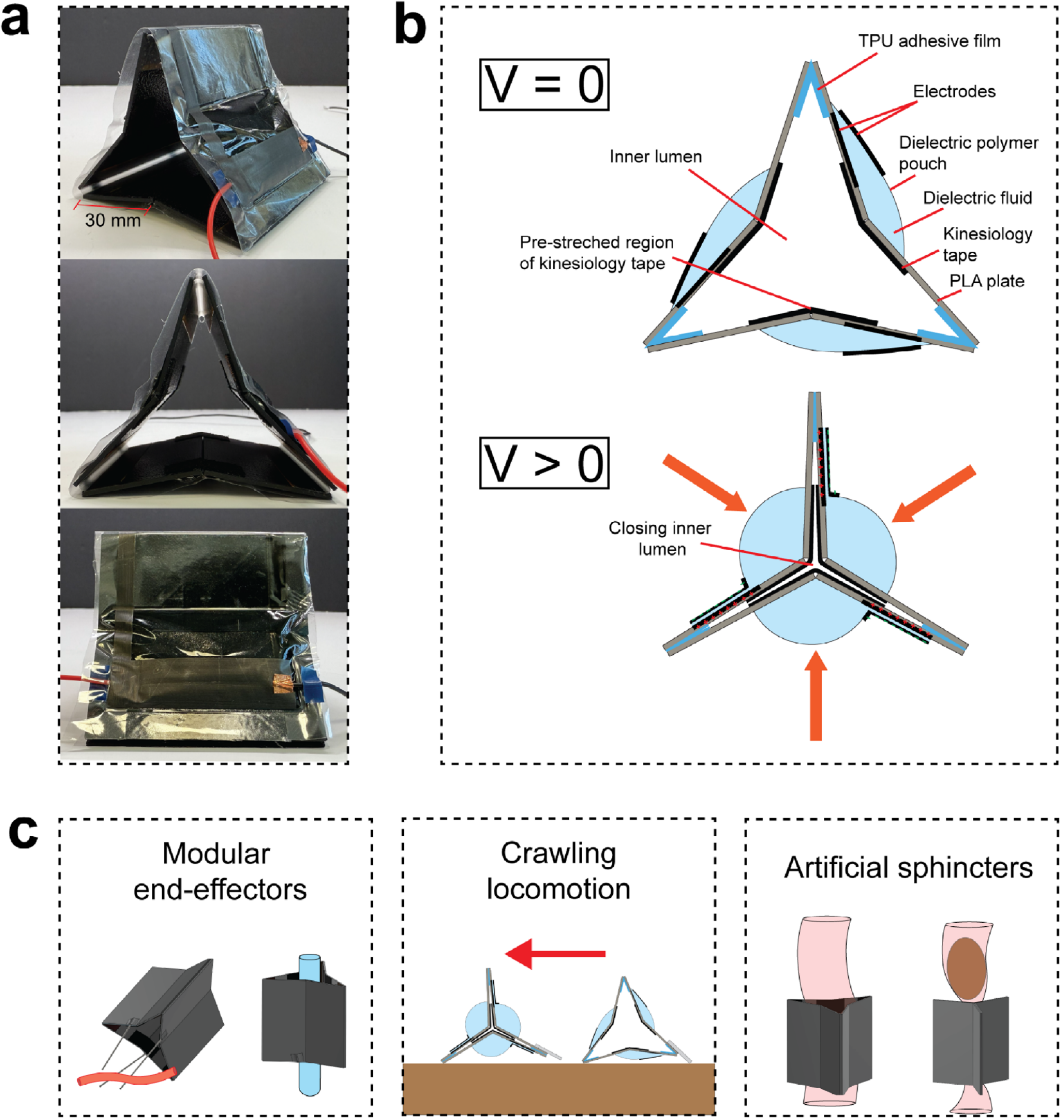
Design and working principle of the EFRA. (a) Images of the EFRA. (b) Labeled diagram and actuation principle of the EFRA. (c) Potential applications of the EFRA. (1) The closing structure of the EFRA with mounting points designed into the skeleton enables its use as robotic end‐effectors with modular attachments for grasping a variety of objects. (2) Attachment of a friction pad also allows the EFRA to crawl, enabling terrestrial locomotion applications. (3) The closing lumen of the EFRA enables its use as an artificial sphincter, preventing the movement of solids from moving through.

This study therefore presents an Electrohydraulic Folding Ring Actuator (EFRA) (Figure 1a,b) that comprises three bending electrohydraulic joints arranged in a ring connected by flexible linkages. Using folding actuation driven by electrohydraulic bending segments to close the inner lumen of the ring as shown in Figure 1b, the EFRA achieves greater efficiency in closure of the ring at a given actuation voltage as compared to existing methods that utilize linearly contracting elements. Using bipolar square wave voltage actuation, the EFRA is capable of achieving near‐full sustained inward radial contraction of 0.89, much greater than the theoretical maximum of 0.59 using linearly contracting methods [27]. This high radial contraction enables the EFRA to be used in manipulation and artificial sphincter applications (Figure 1c) as demonstrated in this work, while maintaining a relatively compact size. Furthermore, as the EFRA is an actively closing, radially contracting actuator that constricts its inner lumen under high voltage, it can lift heavier objects than similar but passively closing DEA‐based actuators [2]. The EFRA achieves sustained inward force outputs of up to 0.96 N while having a low mass of ∼25.9 g and can lift more than four times its weight with payloads up to 110.26 g in robotic manipulation tasks. The rigid skeleton of the EFRA, formed by the constraining rigid plates of the electrohydraulic joints, also serves as a mounting interface for a variety of attachments that broaden the application of the EFRA. For example, the addition of blunt needle tips enables precise robotic manipulation, while friction pads applied on the outside of the EFRA exploit the deforming outer profile of the EFRA to enable crawling locomotion (Figure 1c) as also demonstrated in this work. Overall, the EFRAs offer high radial contraction and sustained inward‐directed force in a compact, low‐profile, and lightweight package, expanding the wider use of radially contracting actuators toward new applications.

## 2 Results

### 2.1 Design

The EFRA takes the form of a triangular ring, comprising three connected electrohydraulic bending joints, with each joint linked together by a flexible hinge as shown in Figure 1b. The annular cavity that forms from the linked electrohydraulic joints forms the inner lumen of the actuator that radially contracts when actuated.

Each electrohydraulic joint is comprised of a dielectric polymer pouch containing a dielectric fluid, with flexible electrodes applied to both sides of the pouch as shown in Figure 1b. Two rigid constraining plates are applied to the pouch to constrain one side of the pouch to provide both support and selective deformation [42]. The rigid plates also function as a skeleton for the actuator, giving it structure. A pre‐stretched elastic tape is also applied across the hinge to increase the restoring force of the actuator in a lower profile method compared to existing methods [42] to prevent excessive occlusion of the inner lumen of the EFRA that will obstruct radial contraction.

When a high voltage is applied across the electrodes of the electrohydraulic joints, the electrodes are induced with a positive and negative charge that induces an electric field across the dielectric polymer pouch layers and the dielectric fluid [38, 40] that also acts to increase dielectric permittivity through the medium between the two electrodes [44]. This results in a Maxwell stress that causes the electrodes to progressively zip together beginning from the tapered edge of the electrodes due to the tapered edge having the highest electric field [45]. This displaces the dielectric fluid toward the non‐electrode‐covered actuating portion of the dielectric pouch, which pressurizes the actuating portion of the pouch. Due to the deformation constraints provided by the two rigid plates applied on each electrohydraulic joint, the pressurization of the fluid at the joint causes joint flexion [42]. As all three electrohydraulic joints in the EFRA are actuated together, further constrained by the flexible linkages applied across each joint, they bend inward, pushing the joint vertices toward the center of the ring. This occludes the inner lumen of the ring, providing radial contraction of the inner lumen of the ring with inward‐directed force. This also further stretches the elastic element applied across the rigid plates of each joint. Due to the bending angle of the electrohydraulic joints being limited to 71.7° (Figure S1), three electrohydraulic joints are used to make the EFRA, as increasing the number of joints would cause a reduction of contraction ratio and a reduction in the ability of the EFRA to achieve full closure.

When the high voltage is turned off, the pre‐stretched elastic element provides a restoring force that pulls the two rigid plates of each electrohydraulic joint back into its rest state, therefore increasing the size of the inner lumen of the actuator back to its initial state as well. The pre‐stretch of the elastic tape also provides an initial tension force that allows the actuator to be placed on its side without collapse, as shown in Figure 1a.

### 2.2 Fabrication of the EFRA

**FIGURE 2:**
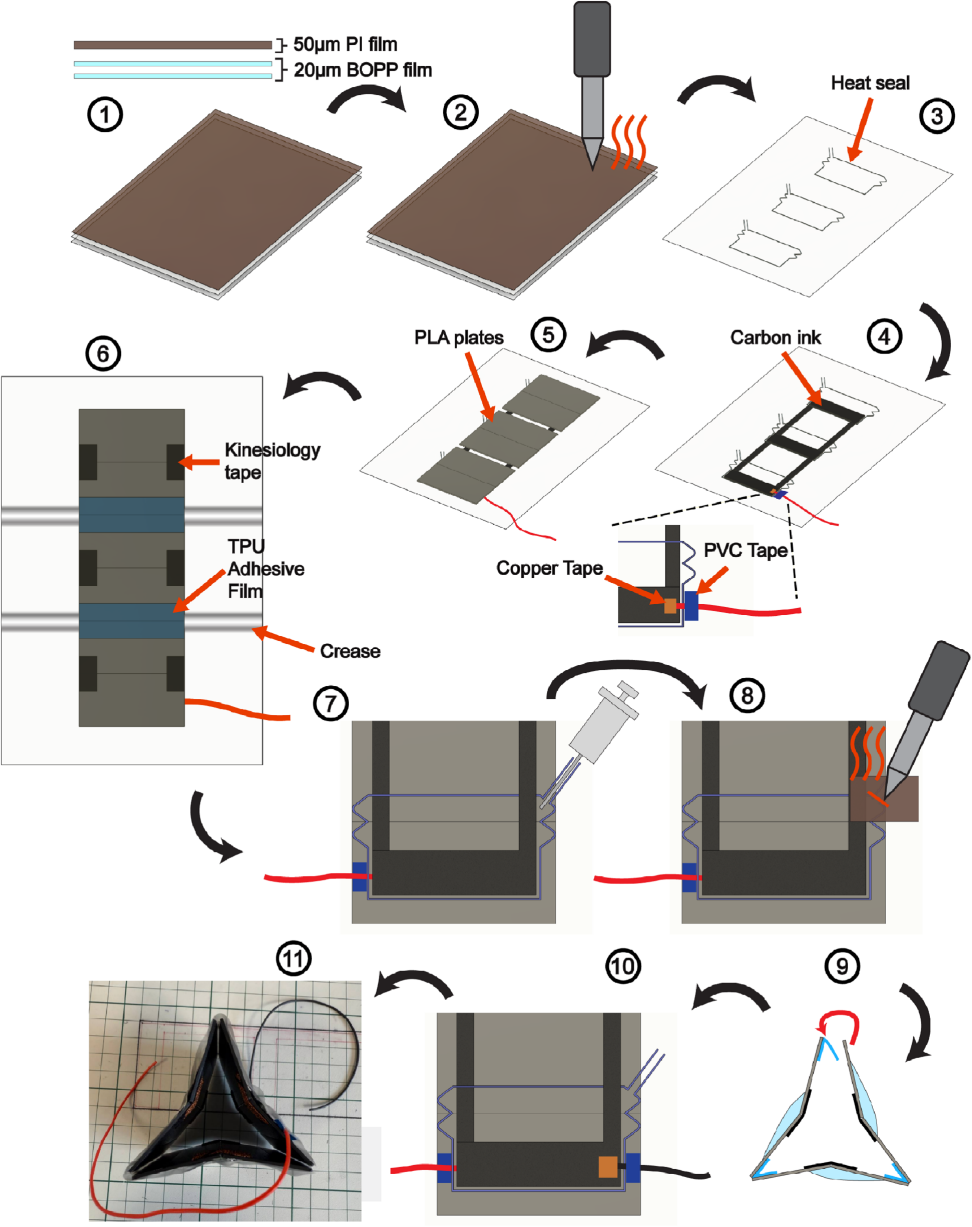
Steps for fabrication of the EFRA. (1) Place 2 sheets of 20µm biaxially oriented polypropylene (BOPP) film underneath a sheet of 50µm PI on the bed of the CNC heat sealer (Figure S2). (2) Heat seal to the shape of the electrohydraulic pouches with the CNC heat sealer. (3) Remove PI film from heat‐sealed BOPP films. (4) Apply carbon ink electrodes to both sides of the sealed BOPP using vinyl transfer tape masks and brush painting. Apply the first wire to the electrode with copper tape, and PVC tape to act as strain relief. (5) Apply rigid PLA plates with double‐sided adhesive. (6) Apply the flexible joints (TPU adhesive film) and elastic elements (kinesiology tape) to the PLA plates. (7) Inject dielectric fluid into each dielectric pouch with a syringe and blunt needle. (8) Seal the fill port of the dielectric pouch with a soldering iron, covered by a piece of 50µm PI film. (9) Fold into a triangular ring and apply TPU adhesive film as the final flexible hinge. Trim off excess BOPP protruding from the perimeter of the rigid PLA plates. (10) Apply the second wire to the outer electrode with copper tape and PVC tape. (11) Final structure of EFRA after fabrication.

Fabrication of the EFRAs as shown in Figure 2 build upon previously developed processes in making HASEL actuators and SES joint‐type actuators [40, 42, 43, 45]. A sheet of three notched electrohydraulic pouches is first created by heat‐sealing 2 20 µm‐thick biaxially oriented polypropylene (BOPP) pouches together with a modified CNC pen plotter machine equipped with a soldering iron set to 225°C. BOPP is selected as the dielectric film due to its ease of heat sealing due to its relatively low melting point to facilitate rapid prototyping, as well as its status as a well‐established dielectric polymer used in HASEL actuator fabrication [27, 38, 41, 45]. A sheet of 50 µm polyimide (PI) film is used to protect the BOPP from thermal damage from the soldering iron, and a 1.5 mm silicone sheet is placed underneath the BOPP to spread out the heat applied by the soldering iron [45]. To apply the electrodes, a vinyl transfer tape mask is cut to the shape of the electrodes with a computer numeric control (CNC) cutting machine (Silhouette Cameo 4) and adhered to the required positions of the electrodes on the heat‐sealed BOPP sheet. Vinyl transfer tape is chosen for the masks due to its low tack to prevent physical damage to the BOPP during removal. Carbon ink (JW‐001) is then painted onto the BOPP sheets with the mask, and the mask is then removed leaving the carbon ink electrodes in the required shape and position. The carbon ink is then dried in an oven at 55°C for 25 min. This is then repeated on the other side. The positive wire is now adhered to one of the electrodes with a copper tape, and a small piece of polyvinyl chloride (PVC) tape is used as strain relief. Rigid plates of dimensions 30mm × 59.67mm × 1mm are 3D‐printed with polylactic acid (PLA), and two plates are adhered to each electrohydraulic joint with double‐sided adhesive (3M 300LSE), leaving a 5 mm gap between each electrohydraulic joint to ensure sufficient slack for the sheets to be folded into the ring structure later. Each of the electrohydraulic joints are then linked together with thermoplastic polyurethane (TPU) adhesive film (Ise Mount TPU repair patch) cut to 20 mm × 59.67 mm to form flexible linkages, with the extra 5mm of material gently creased downward and the rigid plates pushed together before application of the TPU adhesive film. Elastic kinesiology tape (KT Tape Pro Wide) cut to dimensions 10 mm × 20 mm are applied across the hinges of each electrohydraulic joint, with two applied at both ends of a single joint, and with the weft direction of the kinesiology tape aligned in the direction of bending of the hinge. 3.5 mL of dielectric fluid (sunflower oil) is filled into the pouches with a syringe via a fill port designed in the pouches. Sunflower oil is used due to its low viscosity to ensure minimal viscous effects for high‐speed actuation [46] in addition to its low cost. The fill port is then manually sealed with a soldering iron, also with a 50 µm thick PI film covering the BOPP with a small 1.5 mm thick silicone pad underneath the BOPP. The assembly is then folded to form the triangular ring shape, and another TPU adhesive patch is applied to form the final flexible linkage. The ground wire is now connected to the other electrode with a piece of copper tape, also with a PVC tape strain relief.

### 2.3 Radial Contraction Properties

Performance testing for the EFRA is grouped into two main categories: (1) radial contraction ratio, which is the degree of shrinkage of the inner lumen of the actuator, and (2) radial contraction force, which is the inward‐directed force output of the actuator. The radial contraction ratio is a measure of the constriction performance of the actuator, which is important for applications such as manipulators as it determines the minimum size of the object that the actuator can grip, as well as sphincters that require maximum inward contraction to prevent objects or fluids from moving through. The radial contraction force is crucial for applications that require inward‐directed force output, such as in manipulators to grip heavier objects.

#### 2.3.1 Radial Contraction Ratio

Radial contraction ratio is characterized by the following equation [1, 14]:

(1) $$CR=\frac{A_{lum,0}-A_{lum}}{A_{lum,0}}$$

**FIGURE 3:**
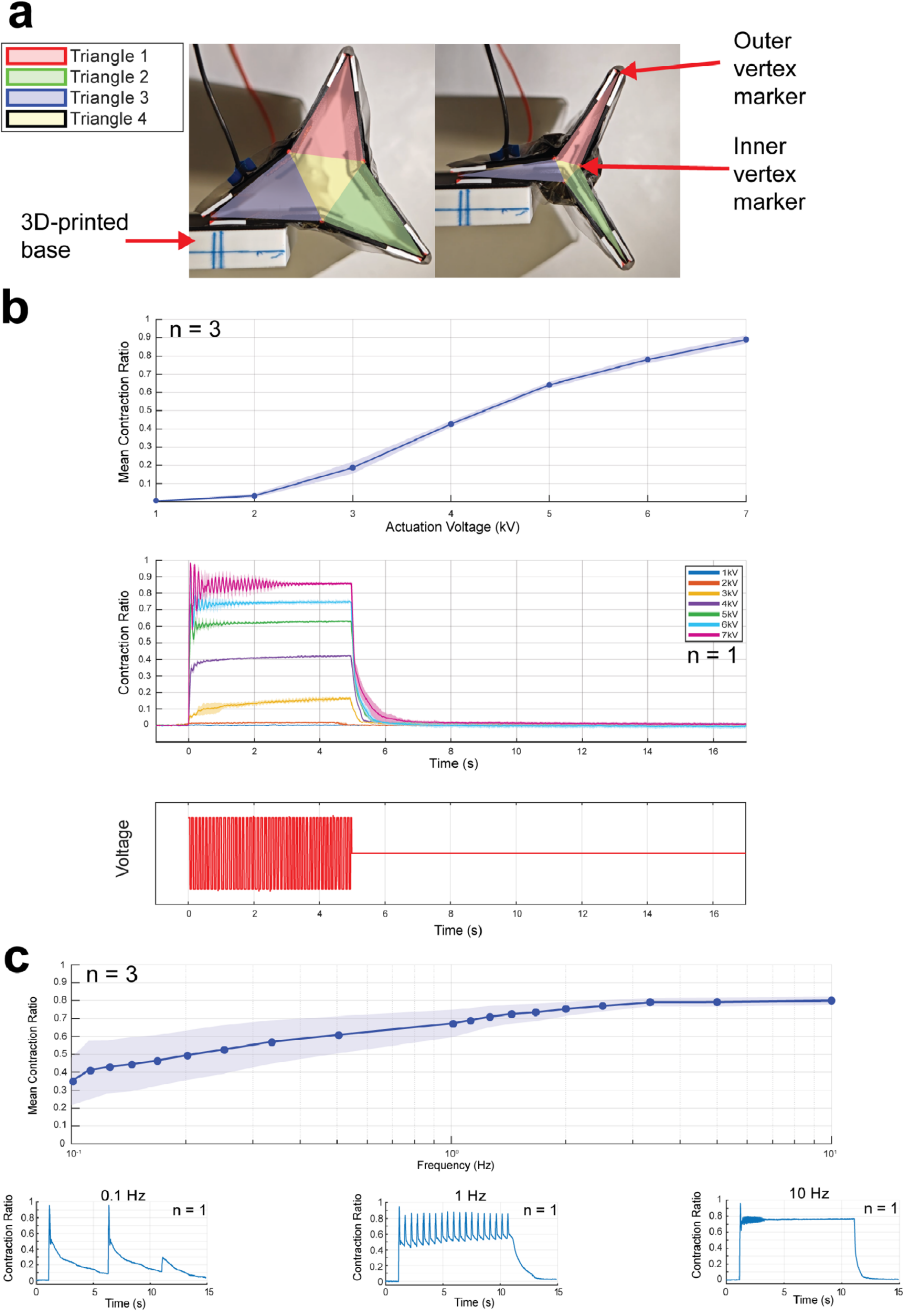
Radial contraction ratio performance of the EFRA. (a) Experimental setup and determination of inner lumen area. (b) Results of EFRA contraction ratio under bipolar square‐wave voltage actuation at 10 Hz. Mean contraction ratio vs actuation voltage is plotted as mean ± SEM across three actuator samples (*n* = 3) with three trials per sample. Contraction ratio vs time is plotted as mean ± SD of one actuator sample (*n* = 1) across three trials. Mean contraction ratio is calculated across 4 s upon actuation onset. (c) Frequency‐dependent actuation stability of bipolar square‐wave voltage actuation at 6 kV, plotted as mean ± SEM across three actuator samples (*n* = 3) with three trials per sample. Contraction ratio vs time plots for 0.1, 1, and 10 Hz utilize data from a single representative trial obtained from Sample 2 (refer to Figure S3).

Where *CR* is the radial contraction ratio, *A_lum,0_* is the initial cross‐sectional area of the inner lumen of the actuator, and *A_lum_* is the cross‐sectional area of the inner lumen of the actuator during actuation. To take the cross‐sectional area of the inner lumen of the actuator into account in the calculation of the radial contraction ratio, the inner lumen of the actuator is taken as four triangles derived from six points on the actuator as shown in Figure 3a: three outer vertices, and three inner vertices. The change in area of these four triangles is then calculated to determine the radial contraction ratio of the EFRA from rest.

Figure 3a also showcases the experimental setup for the radial contraction ratio tests for the EFRA. In this test, the EFRA is mounted to a 3D‐printed test base that acts to suspend the EFRA sideways, to eliminate the effects of gravity on the actuator. To determine the radial contraction ratio of the actuator, small markers made of PVC tape are placed on the three inner vertices and three outer vertices of the actuator, along the edges of the rigid plates forming the skeleton of the EFRA. The actuator is recorded from above with a 240 frames per second (FPS) phone camera (Honor Magic Pro 6) (Movie S1), after which the videos are imported into motion tracking software (Kinovea) [47] to track the position of the markers to calculate cross‐sectional area and therefore radial contraction ratio in MATLAB. To account for the residual area between the markers at maximum contraction (i.e. markers cannot converge to zero area), sample‐specific residual area *A_res_* at full contraction is obtained from the 8 kV unipolar recordings as the mean enclosed area at the frame corresponding to peak contraction across the three trials. The contraction ratio is then computed as:

(2) $$CR=\frac{\left(A_{lum,0}-A_{lum}\right)}{\left(A_{lum,0}-A_{res}\right)}$$

The EFRA was tested with bipolar square wave voltage with a 10 Hz frequency and a 5‐sec duration. Bipolar square wave voltage was selected over unipolar voltage to improve actuation stability and smoothness [48], and mitigate charge retention effects observed in highly insulating films such as BOPP [49], as observed in tests conducted with unipolar voltage as shown in Figure S4. Actuation voltages of 1–7 kV at 1 kV intervals were tested, as 8 kV and above of actuation voltage resulted in observed higher likelihood of dielectric breakdown. Three EFRA samples were tested to observe variability between actuators, with the data of the mean contraction ratio against actuation voltage plotted in Figure 3b. Single actuator contraction ratio against time data also plotted as shown in Figure 3b.

As shown in Figure 3b, actuation with 10 Hz bipolar square waves allowed the EFRA to achieve increased inward contraction stability, with a clear positive relationship between actuation voltage and mean contraction ratio. At 7 kV of actuation voltage, the EFRAs were able to obtain an average of 0.89 across the three samples, which is higher than other HASEL radially contracting actuators [23, 27], although with some underdamping effects as observed in the contraction ratio against time plots for bipolar square wave voltage in Figure 3b, especially at higher actuation voltages. At 6 kV of actuation voltage, the EFRAs were also able to obtain a relatively high radial contraction ratio of 0.78 across the three actuators, which is greater than similar DEA radially contracting actuators that achieved a radial contraction ratio of around 0.73 with passive inward closure of the ring arising from elastic return from its expanded actuated state [2]. Actuator variability was also observed to significantly decrease under bipolar square wave voltage actuation compared to unipolar square wave voltage. This test was however limited to a maximum of 7 kV actuation due to increased observed risk of dielectric breakdown at 8 kV.

At 7 kV, the EFRAs were also noted to have a fast average rise time of 31.48 ms (SEM = 2.45 ms), but an elongated average fall time of 539.35 ms (SEM = 125.21ms). This asymmetry has been previously attributed to dielectric fluid viscosity [42, 50].

The effect of different bipolar voltage frequencies on actuation stability was also investigated to optimize the required frequency for stable and sustained radial contraction. In this test, the EFRA samples were subject to 6 kV bipolar square waves for 10s to obtain the mean contraction ratio across the 10 s duration. This is repeated for a range of increasing frequencies from 0.1 to 10 Hz. It can be observed from Figure 3c that as frequency increases, actuation stability also increases resulting in higher mean contraction ratios, with 10 Hz offering the most stable displacement of the tested frequencies. It is also noted that at higher frequencies, the EFRAs tend to have faster return to their rest configuration when the voltage is returned to zero.

Variability between actuators is shown to be much larger at low frequencies, possibly due to varying dielectric relaxation times as a result of small differences between the polymer films used across different samples, or inconsistent electrode thickness during manual brush‐painting of the electrodes, causing variances in the RC time constant. This is further highlighted in Figure S3, where Sample 1 is shown to exhibit much slower loss of contraction ratio as opposed to Samples 2 and 3. Nevertheless, using higher frequencies such as at 10 Hz substantially mitigates variability between actuators while improving actuation stability (Figure S3).

#### 2.3.2 Radial Contraction Force

The contraction force of the EFRAs is characterized in two different ways: (1) blocked force, where the inward‐directed force output of the EFRAs is measured with the EFRA constrained to zero contraction ratio, and (2) force with displacement, where the force output of the EFRAs is measured with a moving force sensor to allow the EFRA to radially contract, in order to determine the relationship of its force output with different levels of radial contraction.

##### 2.3.2.1 Blocked force

**FIGURE 4:**
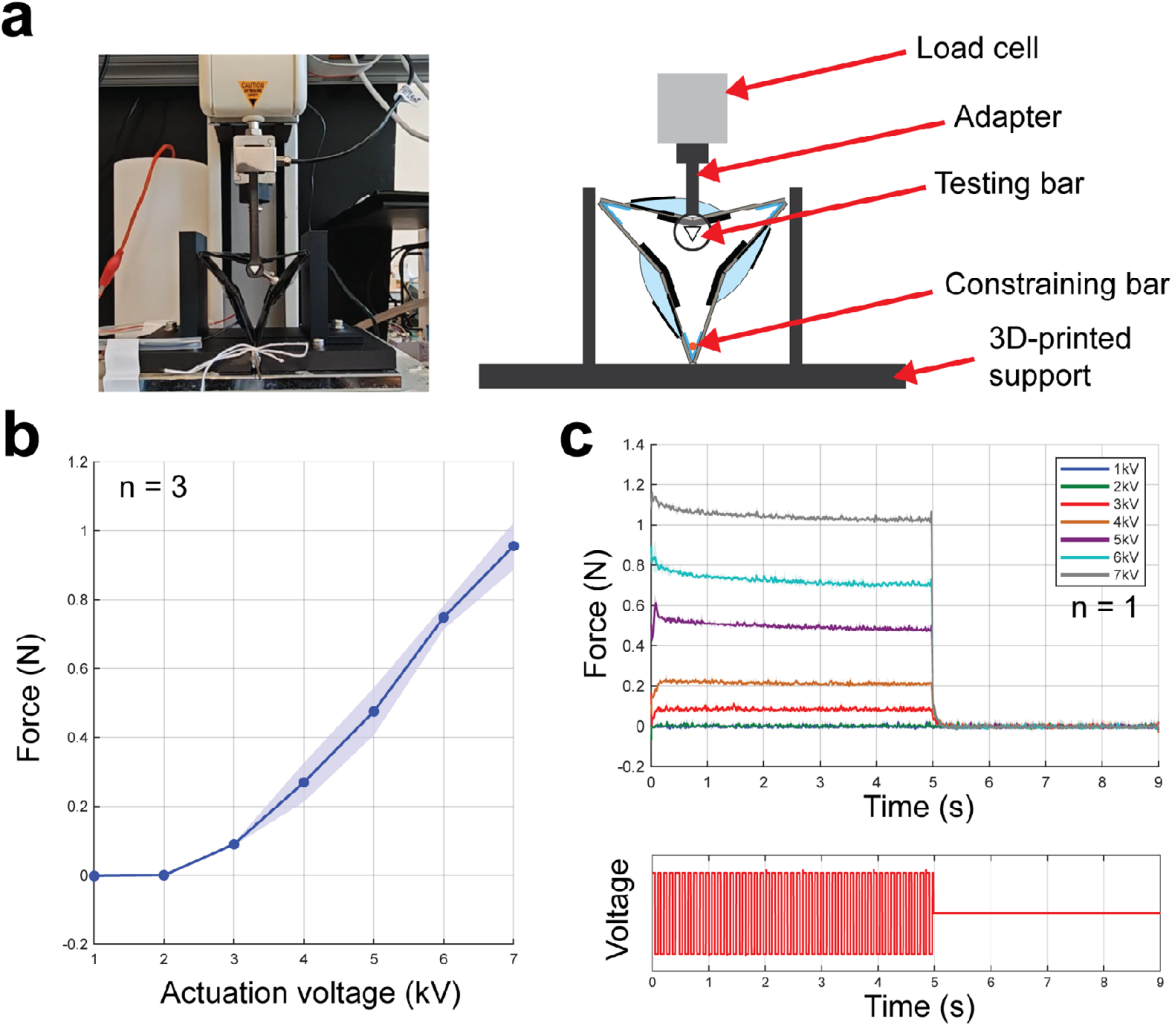
Blocked force performance of the EFRA. (a) Experimental setup. (b) Blocked force vs actuation voltage plotted as mean ± SEM across three actuator samples (*n* = 3), with three trials per sample. Blocked force is determined from the mean force calculated over a 5 s period upon actuation onset. (c) Blocked force vs time plotted as mean ± SD for one actuator sample (*n* = 1) across three trials, with the corresponding voltage waveform shown from a single representative trial.

As shown in Figure 4a, the blocked force test involves the attachment of a small triangular testing bar placed underneath one of the inner vertices of the actuator. The testing bar is attached to an adapter that is in turn attached to a 1 kg load cell (Zhongnuo ZNLBS‐VI, 1kg) to measure the force output of one inner vertex of the EFRA. The load cell is also connected to a 1 kg force gauge (Algol HF‐1 10N Handy Force Gauge) attached to a JSV‐H100 test stand to allow for fine control of the vertical position of the testing bar. 3D‐printed supports are also placed on either side of the actuator such that they barely touch the actuator but help to keep it in its upright position in the event the actuator loses contact with the testing bar. Finally, a thin brass rod and string are used to constrain the actuator to the 3D‐printed base of the setup. This setup constrains inward radial contraction of the actuator while allowing for the recording of the force output of the topmost inner vertex of the actuator. During each trial, average force output over the course of the 5 s actuation was recorded by the load cell with a sampling rate of 1000 Hz, with the force gauge only being used to locate the point at which the testing bar contacts the inner vertex of the EFRA.

The controls of the test stand were first used to raise the testing bar until just before a force reading is observed on the force gauge, to ensure that the testing bar accurately records the blocked force output of the EFRAs while also not pulling the EFRA outward. The blocked force was tested at from 1 to 7 kV of actuation voltage at 1 kV intervals, using bipolar square waves at a frequency of 10 Hz, with three trials per actuation voltage. After each trial, the testing bar is moved upward by 2 mm to pull the EFRAs slightly outward to pressurize the electrode‐covered part of the dielectric pouches to remove any residual adhesion between the electrodes and fully reset the actuator, after which the testing bar is moved back to its original position for the next trial. Three EFRA samples are also tested to obtain data on actuator variability, with the data plotted in Figure 4b, and with single actuator force vs time data showcased in Figure 4c.

As observed from the results shown in Figure 4b, starting from 2 kV onward, all three tested EFRA samples exhibit a positive relationship between actuation voltage and blocked force starting from 2 kV. At 7 kV of actuation voltage, a mean blocked force of 0.96 N was obtained. In the force‐time plot of Figure 4c, higher voltages are also shown to have higher initial peak forces, while lower actuation voltages begin with lower initial forces. Nevertheless, in both cases, force output rapidly converges to a stable value, with clear scaling between steady‐state force and actuation voltage. Upon voltage removal, force rapidly falls and returns to baseline, which is consistent with the contraction ratio with time plots under bipolar actuation voltage shown in Figure 3b.

##### 2.3.2.2 Force with Displacement

As HASEL‐based soft actuators generally exhibit a monotonic decrease in force output with increasing strain [41], we also investigate the decrease in force output of the EFRAs with increasing radial contraction strain.

**FIGURE 5:**
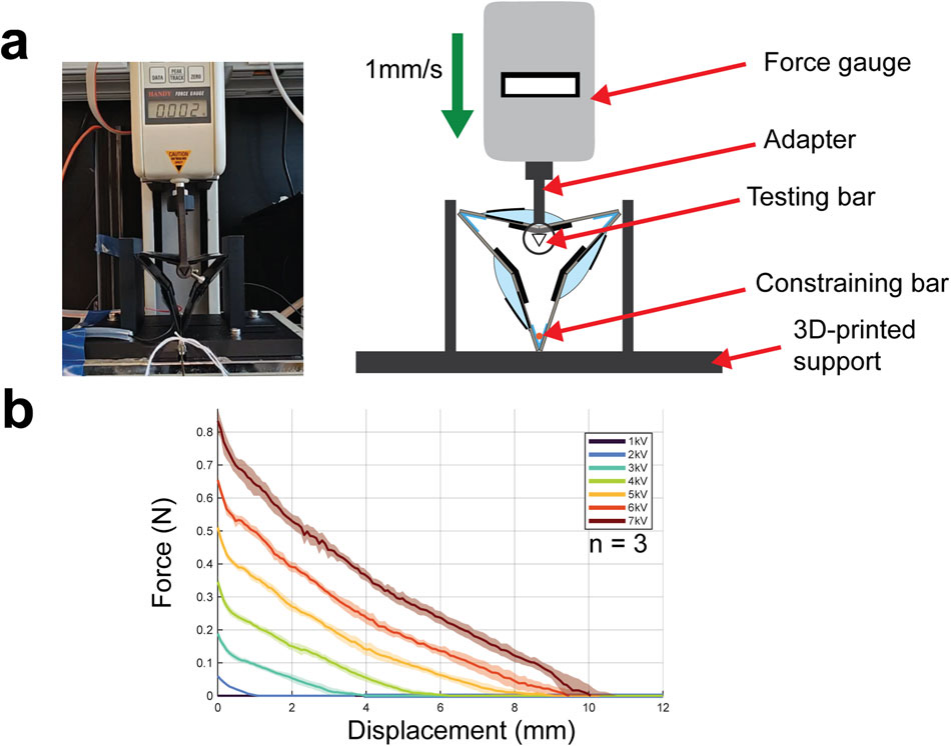
Force vs. displacement performance of the EFRA. (a) Experimental setup. (b) Force vs displacement plotted as mean ± SEM across three actuator samples (*n* = 3), with three trials per sample.

The force with displacement tests utilize a similar experimental setup as the blocked force tests, but with the 1 kg load cell removed, and the testing bar and adapter directly attached to the 10 N force gauge as shown in Figure 5a. Seven actuation voltages were also tested, from 1 to 7 kV with 1 kV intervals. Three trials were also conducted for each actuation voltage. Three actuator samples were also tested to showcase actuator variability. During each trial, the test stand controls were used to move the testing bar just before contact is made with the inner vertex of the EFRA, which was demarcated as the zero‐displacement point. Voltage is then turned on to allow the EFRA to stabilize for 5 s with the testing bar kept stationary to remove the brief initial force instability observed in the blocked force tests. After this stabilization period, the voltage is kept on, and the testing bar mounted to the force gauge is then moved downward at a constant speed of 1 mm/s until the point where the inner vertex of the actuator has lost contact with the testing bar, which indicates the maximum radial contraction reached for that actuation voltage. In this test, the 3D‐printed supports on either side of the actuator serve to prevent the actuator from toppling when the inner vertex loses contact with the testing bar, thereby preventing the actuator from recontacting the bar resulting in erroneous force readings. In between trials for each sample, the testing bar is moved upward from the zero‐displacement point by an extra 2 mm to slightly pull the EFRA outward to fully reset the actuator before each trial.

The results for the force with displacement tests across the three actuator samples are shown in Figure 5b. All actuation voltages showcase a clear decreasing force output with displacement, with the exception of 1 kV which had negligible force output throughout the test. At each actuation voltage at 1 kV and above, an initial peak in force output was generally observed at zero displacement, which then gradually decreases as displacement increases. It is noted that force output generally increases with actuation voltage across the entire displacement range, as indicated by the upward curve shift as actuation voltage increases, with distinct separation between curves. This reflects increasing force output at all levels of radial contraction beyond just blocked force as shown in Figure 4.

### 2.4 Quasi‐Static Model

A quasi‐static model was developed for future actuator optimization, building upon previously developed Helmholtz free energy models for electrohydraulic bending actuators [42, 43]. The free energy model simplifies the EFRA geometry to a single bending unit within the model as per previously developed models, while incorporating additional elastic energy terms due to the kinesiology tape elastic element, as well as the flexible TPU linkages. Additionally, the model outputs are then scaled to the full 3‐bending unit contracting ring structure to estimate contraction ratio and force output performance for comparison with experimental results.

#### 2.4.1 Free Energy Minimization

**FIGURE 6:**
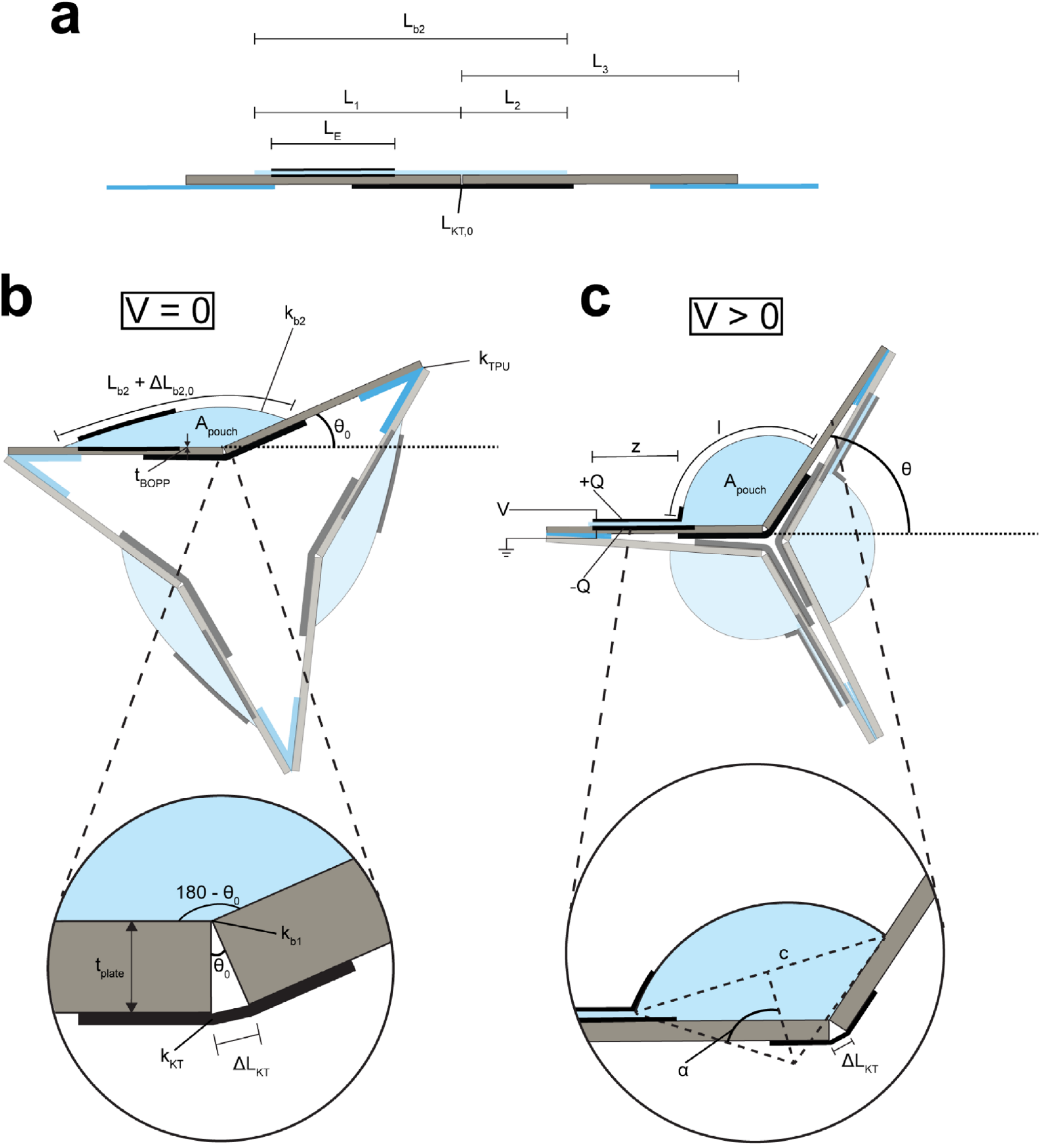
Model parameters for a single bending segment within an EFRA. (a) Single unfilled bending segment. The other 2 bending segments are not illustrated here for simplicity purposes. (b) Filled and unactuated EFRA. (c) Filled and actuated EFRA.

The actuator is first simplified into a single electrohydraulic bending unit as shown in Figure 6a, with *L_b2_* being the length of the pouch comprising *L_1_* and *L_2_*, which are the distances from the zipping edge of the pouch to the hinge, and from the hinge to the end of the pouch respectively. *L_3_* is the plate length, which is identical for all plates used in the actuator, and *L_E_* is the electrode length.

As the pouch is filled with dielectric fluid of volume *V*, the pouch expands with a cross‐sectional area defined as *A_pouch_*, which is estimated as *V* divided by pouch width *w*, and may also be calculated as follows [42, 43]:

(3) $$\begin{array}{ccc} & & A_{\mathrm{pouch}}=\frac{l^{2}}{8\alpha^{2}}\left(2\alpha -\sin\left(2\alpha \right)\right)\\ & & +\frac{1}{4}\sqrt{{\left(c^{2}+{\left(L_{1}-z\right)}^{2}+{L_{2}}^{2}\right)}^{2}-2\left(c^{4}+{\left(L_{1}-z\right)}^{4}+{L_{2}}^{4}\right)}\; \end{array}$$

where *l* is the length of the expanded cylindrical length of the pouch during actuation as shown in Figure 6c, 2*α* is the central angle of the expanded cylindrical length, *z* is the zipping length of the electrodes, and *c* is the chord length of the expanded cylindrical length of the pouch calculated as follows [42, 43]:

(4) $$c=\sqrt{{\left(L_{1}-z+L_{2}\cos\left(\theta \right)\right)}^{2}+{L_{2}}^{2}\sin{\left(\theta \right)}^{2}}$$

The top BOPP film is modeled as a linear spring which extends slightly by a length of *ΔL_0_* when the pouch is filled, with a spring constant of *k_b2_* which is treated as a fitting factor as per previously developed models due to the difficulty of determining *k_b2_* (refer to Supporting Information) [42].

The kinesiology tape elastic element is also treated as a linear spring, with a very small initial distance of *L_KT,0_* as the elastic element is applied across the hinge before pouch filling, and this distance increases to *ΔL_KT_* as shown in Figure 6b after pouch filling. *ΔL_KT_* can be determined using the law of cosines as:

(5) $$\Delta \;L_{KT}=\sqrt{2{t_{plate}}^{2}\left(1-cos\theta \right)}$$

where *t_plate_* is the thickness of the rigid plates.

The spring constant of the elastic element, *k_KT_*_,_ is calculated using an experimentally determined Young's modulus in the weft direction (Figure S5). Small delamination of the elastic element from the rigid plates and a non‐zero inter‐plate gap introduce uncertainty in the initial effective length. As a result, *L_KT,0_* is constrained to be non‐zero and is treated as a fitting parameter to capture the effective initial length of the assembled elastic element, as further described in the Supporting Information.

The hinge of the electrohydraulic joint formed by the bottom BOPP film is treated as a rotational spring of spring constant *k_b1_* [42], calculated based on the typical Young's modulus of BOPP of 2.5 GPa [51], and Poisson's ratio of 0.3 [52]. Finally, the flexible TPU hinge is also treated as a rotational spring with a spring constant of *k_TPU_*, which is also calculated based on an experimentally determined Young's modulus of the TPU adhesive film (Figure S5).

As a voltage is applied across the electrodes, the electrodes are now charged with a magnitude of *Q*, with free energy of the voltage source being −*QΦ*, and the electrodes zip along a length *z*, and the bending angle changes to *θ*. As the dielectric liquid is incompressible [42, 43], cross‐sectional area *A_pouch_* remains constant, and the expanded cylindrical length of the pouch is now a length *l*, where *z + l = L_b2_ + ΔL_b2_*, and where *ΔL_b2_* is given by [42, 43]:

(6) $$\Delta L_{b2}=\frac{c\alpha }{\sin\left(\alpha \right)}+z-L_{b2}$$

As such, the free energy of a single electrohydraulic bending unit of the EFRA as follows prior work [42, 43] but with additional terms for the elastic kinesiology tape elements and TPU adhesive film flexible linkages, is represented as:

(7) $$\begin{array}{ccc} F\left(Q,\;z,\;\alpha ,\;\theta \right) & = & -Q\Phi +\frac{1}{2}Q^{2}\frac{2t_{BOPP}}{\varepsilon_{0}\varepsilon_{r}wz}+\frac{1}{2}k_{b1}\theta^{2}+\frac{1}{2}k_{b2}\Delta L_{b2}{\left(\alpha ,\;z\right)}^{2}\\ & & +\frac{1}{2}k_{KT}\Delta L_{KT}{\left(\theta \right)}^{2}\;+\frac{1}{2}k_{TPU}\gamma {\left(\theta \right)}^{2} \end{array}$$

where *γ* is the bending angle of the TPU film forming the hinge from its application across two adjacent flat plates after which it is folded into its initial angle in the EFRA. Since this angle *θ_h_ =* 60° − *θ* (Equation S2), γ can be written as:

(8) $$\gamma =120^{\circ }+\theta$$

#### 2.4.2 EFRA Modeling

**FIGURE 7:**
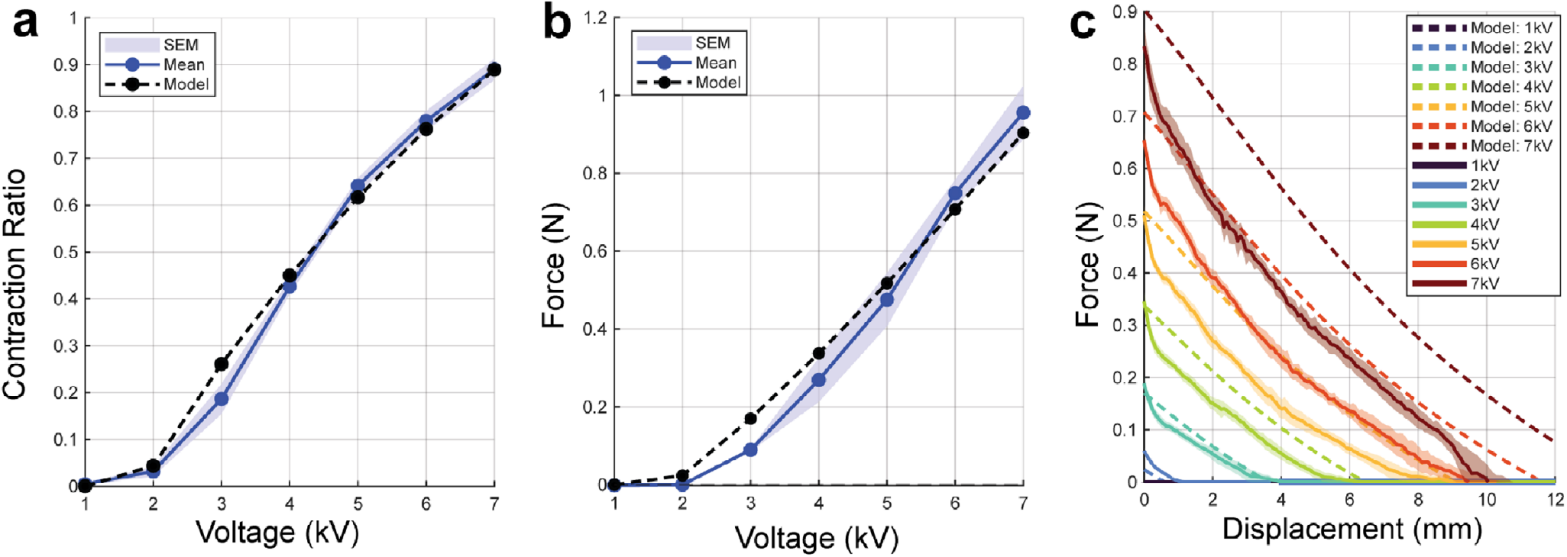
Comparison of quasi‐static model results with experimental data. (a) Comparison of contraction ratio with voltage results using bipolar voltage to model results. (b) Comparison of blocked force with voltage results at 1 inner vertex of the EFRA to model results. (c) Comparison of force with displacement results at 1 inner vertex of the EFRA to model results.

The model is then extended to the full EFRA structure. To model the contraction ratio performance of the EFRA, the relationship between bending angle θ and contraction ratio of the EFRA is first geometrically determined (see Supporting Information and Figure S6). The model results relating θ with voltage are then used to generate the model curve for contraction ratio against voltage as shown in Figure 7a, with comparison against experimental data. Notably, the experimental data used for comparison is the data for bipolar voltage actuation instead of unipolar voltage actuation, due to the rapid displacement loss observed in the unipolar voltage actuation tests not allowing for accurate comparison against quasi‐static Modeling.

To model blocked force output, the blocked torque output τ of a single bending segment is determined by using the virtual work principle, where $\tau =-\frac{\delta F}{\delta \theta }$ for an infinitesimal angular step *δθ* (see Supporting Information). The force output of the hinge of one bending segment is then calculated which corresponds to the force output at one inner vertex of the EFRA. It is noted that the effect of the other two bending segments of the EFRA is assumed to be non‐additive to the force output at the inner vertex of the EFRA and is solely acting as a geometric constraint acting on the tips of the modeled electrohydraulic bending segment (Figure S7). The model results are shown in Figure 7b for comparison against the blocked force results, and Figure 7c for comparison against the force with displacement tests.

The model results closely follow those of the contraction ratio with voltage, as well as blocked force with voltage experimental results as shown in Figure 7a,b respectively, with the exception of slight overestimation of performance at lower voltages and slight underestimation of performance at higher voltages. Notably, the model tends to overestimate the force output of the inner vertex of the EFRA with increasing displacement, especially at higher actuation voltages as displayed in Figure 7c. This is possibly due to the increasing angles that the BOPP hinge, and especially the TPU hinge (see Equation 8) must bend as θ increases, resulting in nonlinear performance of the material at the hinge. As actuation voltage increases to higher values such as 6 or 7 kV and as the contraction ratio reaches near full contraction, there is more deviation from the original spring constant especially for the TPU film forming the flexible hinge, resulting in reduced force output near full contraction.

### 2.5 Applications

The low‐profile design of the EFRAs, combined with their ability to provide sustained high inward radial contraction with inward‐directed force output, provides them with flexibility to be used in various scenarios, in contrast with many existing radial contracting actuators that have designs specific to only one application. The following section will detail some of the possible applications for EFRAs.

#### 2.5.1 Modular End‐Effector

Many radially contracting actuators with robotic manipulation applications typically can only manipulate objects of a particular size or weight class. For instance, while pneumatic‐powered radially contracting actuators can exert high inward‐directed forces for lifting of heavy objects, limited inward radial contraction ratio, actuator geometry restrictions such as a residual large inner lumen gap at full contraction, or lack of fine‐grasping design allowances prevent them from lifting smaller items that require more precision [12, 13, 14, 16]. Electrostatic‐based actuators such as DEA‐based radially contracting actuators may also be limited in their passive force output despite their high payload‐to‐weight ratio of 5.3 due to their low weight, with limited inward radial contraction ratio, lack of active inward‐directed force, as well as lack of fine‐manipulation capabilities due to their design [2].

**FIGURE 8:**
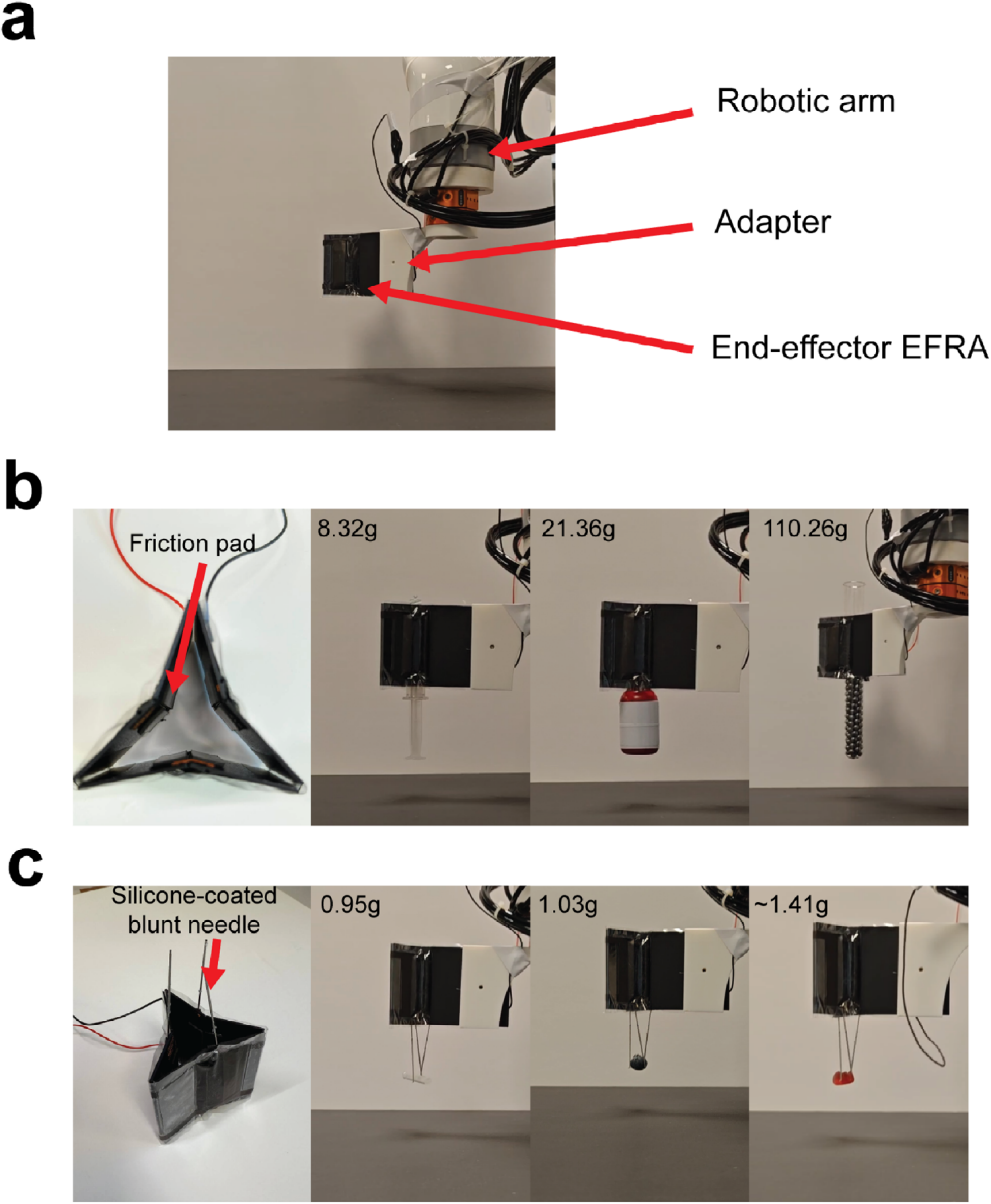
Robotic end‐effector application of the EFRAs. (a) EFRA end‐effector mounted to a robotic arm (Jaka MiniCobo). (b) Coarse gripping configuration of the EFRA end‐effectors. Objects manipulated are a syringe half‐filled with liquid (8.32 g), a small bottle (21.36 g), and a glass test tube containing stainless steel ball bearings (110.26 g). (c) Precision gripping configuration of the EFRA end‐effectors. Objects manipulated are a thin silicone tube (0.95 g), a small blueberry (1.03 g), and a gummy bear (∼1.41 g).

In contrast, the EFRAs not only provide inward force output of 0.96 N for gripping objects within its inner lumen, but combined with the skeleton formed by the rigid plates of each electrohydraulic joint, is also able to provide an end effector platform where attachments can be rapidly configured onto the EFRA using the rigid compliant skeleton as attachment points. To demonstrate the modular end‐effector application of the EFRAs (Movie S2), we design an adapter to allow for attachment of an EFRA to a robotic arm as shown in Figure 8a. We attach 6 adhesive silicone pads along the inner lumen of the EFRA as shown in Figure 8b to enable coarse gripping of objects that fit within its inner lumen. Additionally, for the EFRAs to grab small items or long and thin items that may not fit within the lumen of the actuator, we design additional small attachment points within three of the 3D‐printed rigid plates of the EFRA as shown in Figure 8c, within which silicone‐coated blunt needle attachments can be rapidly installed by simple friction‐fitting to provide precision‐gripping functionality to the EFRA end effectors, and which can be easily removed to revert the EFRA end effectors to its coarse gripping configuration.

As shown in Figure 8b, the coarse grip configuration of the EFRA end effectors was able to grip a variety of objects of varying masses, with the heaviest being a test tube containing stainless‐steel ball bearings which could be loaded to a maximum total mass of 110.26 g before slippage occurred.

In Figure 8c, the precision grip configuration is shown to also be able to manipulate very small objects such as a single blueberry or a gelatin‐based gummy bear without damage to the object. Furthermore, the precision‐grip style was also shown to be able to grip long and thin objects such as a thin silicone tube that would be otherwise impossible to fit within the lumen of the EFRA in its coarse grip style.

It is however noted that the smaller the object, the smaller the payload that the end effector is able to lift, due to the decrease in inward‐directed force output at higher levels of radial contraction as shown in Figure 5b, with maximum payload being attainable when the object constrains the EFRA in its blocked configuration.

#### 2.5.2 Friction‐Based Crawling Locomotion

The deforming outer profile of the EFRAs also provides possibilities for crawling locomotion. The fast response of electrohydraulic actuators allows for the design of crawling robots that can crawl across a surface with high velocities [53].

**FIGURE 9:**
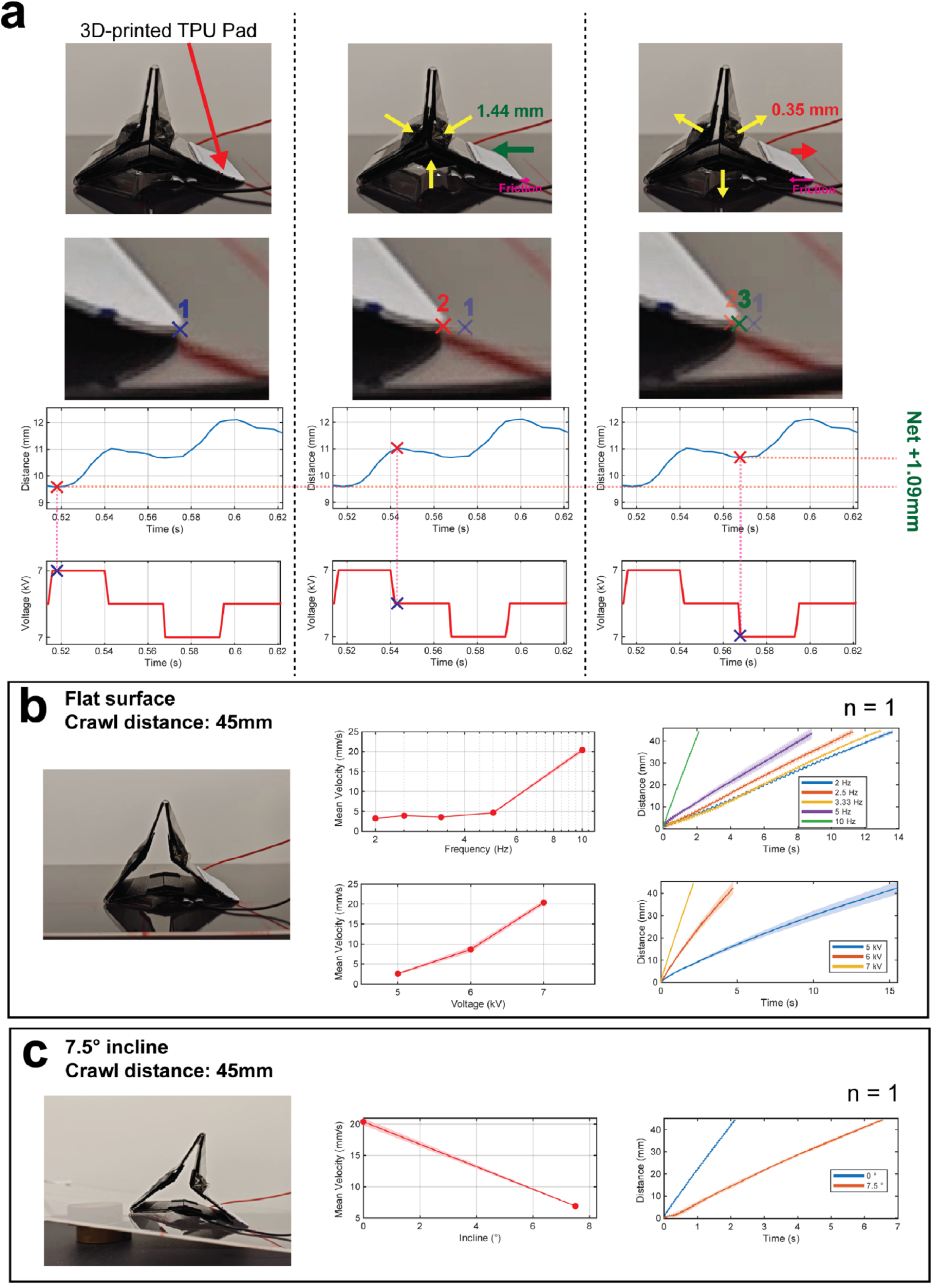
Crawling application of the EFRA. (a) Principle of the crawling EFRA, with displayed step cycle. An illustrative single‐step displacement from an additional demonstration trial with synchronous voltage and displacement tracking demonstrates the net forward displacement per step cycle. The following panels (b,c) report quantitative performance evaluated across three trials from a single actuator sample (*n* = 1) where only kinematic tracking was utilized. (b) Crawling performance on a flat acrylic surface over a 45 mm crawling distance, at various frequencies (2, 2.5, 3.33, 5, and 10 Hz), as well as different actuation voltages (5, 6, and 7 kV), plotted as mean ± SD, with three trials per condition. The data obtained at 7 kV and 10 Hz is used as a shared reference point across the corresponding frequency, voltage, and incline plots. (c) Crawling performance on an acrylic surface at 0° and 7.5° inclines, plotted as mean ± SD, with three trials per incline.

In this application, we achieve friction‐based crawling locomotion of the EFRA (Movie S3) by attaching a 3D‐printed TPU friction pad (Diabase Engineering X60) to one of its outer vertices as shown in Figure 9a, with the BOPP at the attachment point removed to allow the friction pad to directly adhere to the rigid plate with a double‐sided adhesive (3M 300LSE). This pad extends outward from the vertex and serves as a “back foot”, contacting the surface in place of the vertex itself. The crawling surface used in this application was a clean acrylic surface. Average velocity calculated in all tests shown in Figure 9 was measured over the first 45 mm of locomotion, although the crawling EFRA was allowed to crawl for a total of around 50 mm (Movie S3). A smartphone camera (Honor Magic Pro 6) was used to record all crawling experiments from the side at 240 FPS, and the crawling velocity was determined through motion analysis in Tracker [54]. Average velocity over the 45 mm distance is finally calculated by linear fit to account for slight variations in overall crawling velocity across the crawling window.

To enable crawling, we apply a bipolar square‐wave voltage with intervals, at a 50% duty cycle [53] as shown in Figure 9a. Upon voltage application, the EFRA undergoes rapid radial contraction, pulling its three outer vertices slightly inward. During this phase, the friction pad momentarily lifts or jumps forward as the EFRA contracts before recontacting the surface. As the voltage is removed during the interval, the EFRA relaxes and expands back outward. At this point, as the friction pad is fully in contact with the surface, the increased friction with the surface prevents it from sliding back as far as it moved forward, resulting in net forward locomotion as shown in Figure 9a. Subsequent steps are repeated using square waves of alternating polarity to mitigate charge retention effects.

To investigate the locomotion properties of the crawling EFRA, we also tested the EFRAs at different actuation frequencies, different voltages, as well as on an incline.

To test the effects of actuation frequency on the velocity of the crawling EFRAs, the crawling EFRA was also subject to bipolar square‐wave actuation voltage at an amplitude of 7 kV with regular intervals at 50% duty cycle, but with 5 different frequencies of 2, 2.5, 3.33, 5, and 10 Hz, and three trials conducted per frequency. The results as shown in Figure 9b indicate that increasing frequency has a clear benefit in increasing velocity of the crawling EFRAs, with 10 Hz achieving 20.39 mm/s mean velocity. Combined with the body length (BL) of the crawling EFRA with its added friction pad of around 72 mm, this translates to a crawling velocity of around 0.28 BL/s.

The effect of actuation voltage on the velocity of the crawling EFRA was also tested by subjecting it to two additional actuation voltages; 5 and 6 kV, standardized with bipolar voltage square‐waves with an interval of 10 Hz, and with three trials conducted per actuation voltage. As shown in Figure 9b, increasing voltage also yields a clear improvement in crawling velocity, attributed to higher radial contraction attained at higher actuation voltage resulting in larger steps taken by the crawling EFRA.

The ability of the EFRA to crawl on an inclined surface was also tested. The acrylic surface was inclined by 7.5°, with brass weights placed underneath the acrylic surface to act as a support to prevent the acrylic surface from vibrating from the crawling EFRA's locomotion. Actuation voltage was also set to 7 kV with the same voltage waveform. The results shown in Figure 9c indicate that the crawling EFRA was still able to achieve relatively consistent velocities across the 45 mm of locomotion, but with notably slower mean velocity of 6.92 mm/s or around 0.10 BL/s, attributed to smaller steps being taken by the crawling EFRA as it works against gravity with smaller distances covered by each jump of the friction pad relative to the axis of the crawling plane.

#### 2.5.3 Artificial Sphincter

In contrast to current electrohydraulic sphincters that are designed to model the urethral sphincter to stop fluid flow [23], we instead take advantage of the high inward radial contraction and inward‐directed force output of the EFRAs as a gut sphincter model for more solid objects (Movie S4). In this application, a polyethylene (PE) film sleeve of 38 mm in diameter was fitted within the inner lumen of an EFRA. Application of 7 kV of 10 Hz bipolar square wave voltage to the EFRA causes radial contraction of the EFRA. This constricts the PE sleeve within its inner lumen, blocking objects from passing through.

**FIGURE 10:**
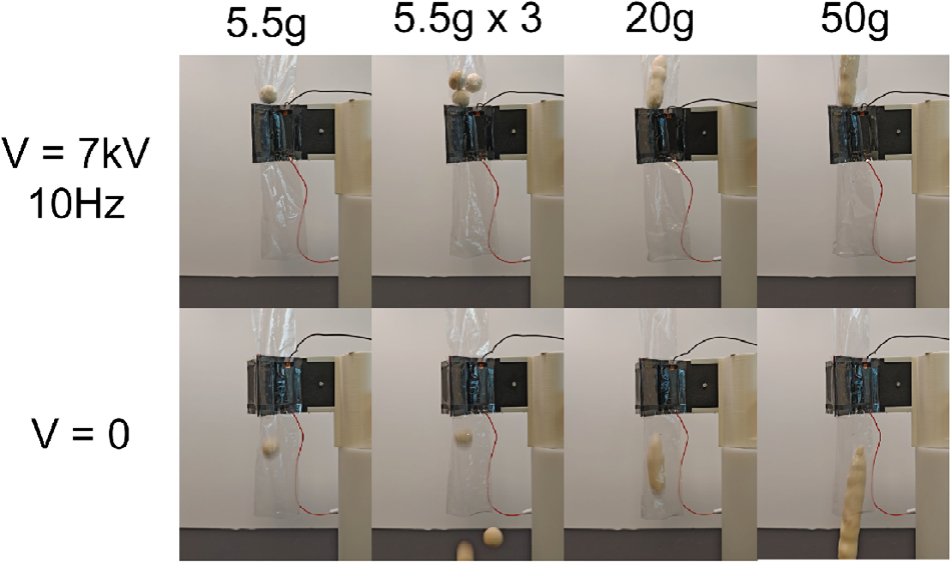
Artificial sphincter application of the EFRA. 4 different scenarios were tested: (1) 1 × 5.5g chyme model, (2) 3 × 5.5g chyme model, (3) 20g chyme model, and (4) 50g chyme model.

To demonstrate this application, we utilize modeling clay of different masses as chyme models (5.5, 20, and 50 g). The EFRA was suspended above a surface with the PE sleeve inserted in its inner lumen. The EFRA is then actuated at 7 kV 10 Hz bipolar square wave voltage to constrict the PE sleeve, and the modeling clay chyme models are dropped into the top opening of the sleeve, where they are then caught by the constricted EFRA. 4 different trials are conducted with different chyme models: (1) one 5.5 g chyme model, (2) three 5.5 g chyme models, (3) one 20 g chyme model, and (4) one 50g chyme model. As shown in Figure 10, the EFRA artificial sphincter successfully blocked the chyme models from falling through the PE sleeve when actuated, and with the removal of voltage and relaxation of the EFRA, the chyme models were able to fall through the inner lumen of the EFRA, dropping out of the bottom of the PE sleeve.

The EFRA artificial sphincter also offers an electrohydraulic alternative to the widely used pneumatic or cable‐driven actuation seen in many soft robotic sphincters [55, 56], with the added advantages of the low profile and lightweight design of the EFRA. In contrast to existing HASEL‐based sphincters that are designed for urethral simulation through compressing a thick‐walled urethra to halt fluid flow [23], the higher radial contraction of the EFRA artificial sphincter provided by the folding closure of the ring, combined with the structural rigidity provided by the skeleton of the EFRA, allows for the stoppage of solid objects against gravity. While the required high actuation voltages may limit its capacity to be used for in vivo applications, the EFRA artificial sphincter may also be used in other bioinspired robotic designs, or as an ex vivo gut sphincter model such as for simulation of esophageal sphincters, or for simulation of stool movement at the anal sphincter.

## 3 Conclusion

In this work, we have designed a compact electrohydraulic radially contracting actuator that is capable of high inward contraction and occlusion of its inner lumen of up to 0.89 contraction ratio at 7 kV of actuation voltage, with a meaningful inward‐directed force of 0.96 N at 7 kV of actuation voltage. Through leveraging previously developed high‐performance electrohydraulic bending joints [42], we introduced a novel method of achieving high‐performance radial contraction in soft actuators through a folding ring design that translates the rotational motion of individual electrohydraulic bending units into radial contraction, while retaining a compact design to enable versatility in different applications. Through the use of folding actuation, we further achieve greater radial contraction performance than other electrohydraulic radially contracting actuators [23, 27].

As shown in Table 1, the EFRA has a very high contraction ratio of 0.89 compared to DEA and linearly contracting HASEL alternatives that have lower contraction ratios of 0.73 [2] and 0.59 [27] respectively. This high contraction ratio is comparable to and even exceeds some pneumatic variants, except for variants that can reach full radial contraction although with a much bulkier design comprising 5 soft pneumatic actuators expanding into the inner lumen of the ring [1]. The EFRA, being an electrohydraulic actuator, has a much faster actuation speed as opposed to pneumatic soft actuators due to eliminating the need to pump a fluid through a series of tubes and connectors [40, 41, 50], with clear benefits in actuation speed compared to SMA [3] and liquid crystal elastomer (LCE) [32] actuators. Furthermore, the EFRA is also shown to reach full contraction at a much faster speed compared to similar DEA actuators, with a rise time of 31.48 ms compared to the DEA variant [2]. While the EFRA may not be able to lift extremely heavy loads of up to several kilograms that pneumatic actuators are capable of lifting as shown in Table 1, we have demonstrated that the inward‐directed force output of the EFRA is sufficient to carry out manipulation tasks in an end‐effector application with maximum payloads of up to 110.26 g, as well as for constriction tasks as showcased in the artificial sphincter application.

**TABLE 1:** Comparison of RCAs.

Radially contracting actuator	Actuation Type	Deformation type	Radial Contraction Ratio (in area)	Actuation Speed	Force	Note
Soft ring‐shaped actuator [14]	Pneumatic	Expansion	∼0.47 at 15 kPa^a^	Moderate (Pneumatic) ^c^	1 kg gripping payload	
Circular pneumatic muscle actuator (CPMA) [12]	Pneumatic	Linear Contraction	∼0.68 for single actuator ^a^, ∼0.71 for triple actuator ^a^	Moderate (Pneumatic) ^c^	10.9 kg maximum gripping payload for single actuator, 40 kg maximum gripping payload for triple actuator	
Ring‐shaped soft pneumatic actuator (RiSPA) [1]	Pneumatic	Expansion	0.96 symmetrical contraction^a^, ^b^, 1.0 asymmetrical contraction^a^	Moderate (Pneumatic), 3 s settling time	Not reported ^c^	
Fabric‐based star soft robotic gripper [16]	Pneumatic	Bending	∼0.93 ^a^, ^b^	Moderate (Pneumatic) ^c^	∼6 N for single actuator radial contractile force, >8.7 kg maximum gripping payload for triple actuator	
Meshworm [3]	SMA	Linear contraction	∼0.24 ^a^ at 400 mA	Moderate, 0.25s contraction at 400 mA, 2.3–3.6 s relaxation	Not reported ^c^	400 mA causes temperatures above T_g_ of body material, and can only be applied for short periods of time
Circular four‐strand weaving actuator (CFWA) [32]	LCE	Linear contraction	∼0.65 at 0.2A ^a^, ∼0.66 at 0.25 A ^a^	Slow, 90 s to reach 0.66 radial contraction at 0.25 A	∼18 N at 20.5 mm diameter and 0.175 A, ∼4.73 N at 15 mm diameter, and 0.175 A	
Muscle inspired DEA [2]	DEA	Bending	0.73 ^a^, ^b^ (taking expanded state as initial state, and passive contracted state as final state)	Moderate, ∼1.5s for max bending deformation	Passive inward closure, maximum gripping payload of 31g	
High‐strain Peano HASEL [27]	Electrohydraulic	Linear contraction	0.59 ^b^	Fast, electrohydraulic ^c^	2.73 kPa pumping pressure	Reported artificial circular muscle air pumping application at 4 Hz (2.30 L min^−1^). Longitudinally contracting variant achieved peak strain rate of 2176% s^−1^ (0.2N load). Direct radial contraction speed not measured.
EFRA	Electrohydraulic	Folding	0.89	Fast, 31.48 ms rise time, 539.35 ms fall time	1 N, maximum gripping payload of 110.26g	

^a^ Converted from reported values into area contraction ratio

^b^ Calculated based on the smallest inscribed circle that can fit within the actuator

^c^ Metric not measured

We also showcased the versatility of the actuator through presenting different use cases of the actuator in this work, including (1) modular robotic manipulation with rapidly configurable gripping modes for objects of different masses, sizes, and shapes, (2) a locomotion application taking advantage of the deforming outer profile of the EFRA, and finally (3) an artificial sphincter application that utilizes the high inward occlusion of the inner lumen of the actuator. Scientific contributions of this work include (1) the exploration of a novel folding electrohydraulic radially contracting actuator that utilizes bending deformation to achieve radial contraction, (2) comprehensive characterization of the actuator in terms of its contraction ratio and force output performance as well as its frequency‐dependent actuation performance, and (3) mathematical Modeling that builds upon previously developed Helmholtz free energy models, introducing additional effects of passive elastic return mechanisms, and extends the model to estimate performance of a contractile ring actuator.

Future work will involve further tuning and performance improvements to the actuator in terms of its force output, aided by the mathematical model discussed in this work. Further developments in the application of the EFRAs can also be explored, such as extension of the robotic manipulation application to a greater variety of objects through the use of different gripping attachments installed onto the EFRAs combined with potential closed‐loop control for precise gripping through feedback obtained from measuring the capacitance of the actuator due to the self‐sensing nature of HASEL actuators [57].

While each EFRA features a compact design and a low average mass of 25.9g, the EFRAs in this work are powered by a Trek 10/40A‐H‐CE high voltage amplifier which significantly adds to the overall footprint and mass of the entire system. However, other work in literature demonstrates that HASEL actuators can be driven by highly compact driving electronics and batteries [43, 45, 58, 59, 60], removing the need for bulky, benchtop high voltage amplifiers. Integrating similar battery‐powered driving electronics will be a key focus of our future work to improve the versatility and overall design compactness of the EFRA and enable untethered operation. Currently, a primary limiting factor in the choice or design of compact high voltage amplifiers is the requirement for bipolar voltage to mitigate charge retention on BOPP‐based electrohydraulic actuators. Future work will also involve the adoption of dielectric matching and the use of ‘lossy’ polymer films, such as biopolyester [49]. These material improvements will optimize the performance of the EFRA under unipolar voltage, which will also enable the use of simpler DC amplifiers that can be powered by a small low‐voltage battery. Examples include the HVM Technology SMHV05100 that has an output voltage of 10 kV and an input voltage of 5V, with dimensions of only 21.59 mm × 21.59 mm × 15.24 mm, and a typical mass of only 15 g [61].

Besides performance improvements and untethered operation, other future work will be directed toward identifying further applications that take advantage of the high contraction ratio and the inward‐directed constriction force output of the actuator. One potential future application is the development of peristaltic mechanisms based on the EFRA. Biological peristaltic transport mechanisms involve a combination of longitudinal and circular muscles working together to induce a wave‐like motion to transport mass, or chyme, through the gut [7]. As the radially constricting motion of the EFRAs offers an analogue to biological circular muscles, future development will involve the integration of linearly contracting components to simulate the longitudinal muscles to achieve peristalsis. To further facilitate the transport of objects through such a peristaltic transport mechanism powered by the EFRAs, work to downsize the width of the actuators will be conducted to minimize the discrete nature of the peristaltic waves that would result from the current EFRA widths of 59.67mm. It is noted that this would also require mitigating the general reduction in force output of electrohydraulic joints associated with decreasing pouch width [42].

Another future development for the EFRAs is the development of underwater locomotion modules powered by the EFRA. Electrohydraulic or HASEL actuators have been used in previous works to develop high‐performance underwater locomotion robots [59, 60, 62, 63], due to their high performance and versatile nature, allowing for untethered battery‐powered operation without requiring the use of bulky fluidic pumps [59, 60]. As the EFRA is an RCA that acts as a robotic analogue for biological circular muscles, the high radial contraction performance of the EFRA could allow for the development of new methods of electrohydraulic underwater locomotion that swim by jet propulsion, such as in the case of cephalopods [10], and jellyfish [11]. With material and performance optimizations, waterproofing, and the development of untethered battery‐powered electronics as discussed above, the EFRA holds potential for the development of high‐performance electrohydraulic jet propulsion.

## 4 Experimental Methods

### 4.1 Actuator Control

In all experiments, the EFRAs were powered with a high voltage amplifier (Trek 10/40A‐H‐CE, Advanced Energy) connected to a multifunction DAQ (USB‐6211, National Instruments). A/D sampling rate and D/A outputting rate of the DAQ is set to 1000 Hz. The DAQ is connected to a laptop by and controlled via LabVIEW 2018, where parameters to control voltage wave amplitude and frequency are adjusted.

### 4.2 Artificial Intelligence Generated Content Disclosure

The original text of this manuscript is drafted by the authors. Large language models (LLMs) (ChatGPT, Gemini) were used for language editing of the manuscript, and for MATLAB code writing assistance for experimental data processing and for the energy minimization model presented in this work. All text in this manuscript and data processing scripts were thoroughly reviewed by the authors. The authors take full responsibility for all text, as well as all data processed and presented in this manuscript.

## Author Contributions

**Quan Xiong**: conceptualization, methodology, writing – review and editing. **Zeynep Altundal**: writing – review and editing, data curation, investigation. **Gavril Yong En Tan**: software, writing – review and editing, conceptualization, data curation, formal analysis, validation, investigation, visualization, methodology, writing – original draft. **Jonathan William Ambrose**: writing – review and editing, methodology. **Raye Chen‐Hua Yeow**: funding acquisition, project administration, supervision, writing – review and editing, resources.

## Conflicts of Interest

The authors declare no conflicts of interest.

## Supporting information




**Supporting File 1**: advs75613‐sup‐0001‐SuppMat.docx.

**Supporting File 2**: advs75613‐sup‐0002‐MovieS1.mp4.

**Supporting File 3**: advs75613‐sup‐0003‐MovieS2.mp4.

**Supporting File 4**: advs75613‐sup‐0004‐MovieS3.mp4.

**Supporting File 5**: advs75613‐sup‐0005‐MovieS4.mp4.

## Data Availability

The data that support the findings of this study are available from the corresponding author upon reasonable request.
