# Supplementary material for: Electrohydraulic Folding Ring Actuators for Radially Contracting Applications
Source: Adv Sci (Weinh). 2026 May 11:e75613. Online ahead of print. doi: 10.1002/advs.75613 (PMC13335884; doi:10.1002/advs.75613)
Supplement: Supplementary file 1 — Supporting File 1: advs75613‐sup‐0001‐SuppMat.docx. [file ADVS-9999-e75613-s001.docx]

**Supporting Information**

*Determination of model parameters*

To obtain the value of parameters k_b1_ and k_TPU_ which are spring constants for the rotational springs modelled at the BOPP hinge and the TPU hinge respectively, the Young’s modulus and Poisson’s ratio are used to calculate the spring constants. For each hinge, for a width w and hinge length L, the spring constant can be determined by the equation $k=\frac{\mathrm{wD}}{L} ADDIN ZOTERO\_ITEM CSL\_CITATION \{"citationID":"7sktemPT","properties":\{"formattedCitation":"\backslash\backslash super [1]\backslash\backslash nosupersub\{\}","plainCitation":"[1]","noteIndex":0\},"citationItems":[\{"id":1014,"uris":["http://zotero.org/users/14378566/items/8QJ97MJ4"],"itemData":\{"id":1014,"type":"article-journal","abstract":"The impressive locomotion and manipulation capabilities of spiders have led to a host of bioinspired robotic designs aiming to reproduce their functionalities; however, current actuation mechanisms are deficient in either speed, force output, displacement, or efficiency. Here-using inspiration from the hydraulic mechanism used in spider legs-soft-actuated joints are developed that use electrostatic forces to locally pressurize a hydraulic fluid, and cause flexion of a segmented structure. The result is a lightweight, low-profile articulating mechanism capable of fast operation, high forces, and large displacement; these devices are termed spider-inspired electrohydraulic soft-actuated (SES) joints. SES joints with rotation angles up to 70^{\circ}, blocked torques up to 70 mN m, and specific torques up to 21 N m kg-1 are demonstrated. SES joints demonstrate high speed operation, with measured roll-off frequencies up to 24 Hz and specific power as high as 230 W kg-1-similar to human muscle. The versatility of these devices is illustrated by combining SES joints to create a bidirectional joint, an artificial limb with independently addressable joints, and a compliant gripper. The lightweight, low-profile design, and high performance of these devices, makes them well-suited toward the development of articulating robotic systems that can rapidly maneuver.","container-title":"Advanced Science","DOI":"10.1002/advs.202100916","ISSN":"2198-3844","issue":"14","language":"en","license":"© 2021 The Authors. Advanced Science published by Wiley-VCH GmbH","note":"\_eprint: https://advanced.onlinelibrary.wiley.com/doi/pdf/10.1002/advs.202100916","page":"2100916","source":"Wiley Online Library","title":"Spider-Inspired Electrohydraulic Actuators for Fast, Soft-Actuated Joints","volume":"8","author":[\{"family":"Kellaris","given":"Nicholas"\},\{"family":"Rothemund","given":"Philipp"\},\{"family":"Zeng","given":"Yi"\},\{"family":"Mitchell","given":"Shane K."\},\{"family":"Smith","given":"Garrett M."\},\{"family":"Jayaram","given":"Kaushik"\},\{"family":"Keplinger","given":"Christoph"\}],"issued":\{"date-parts":[["2021"]]\}\}\}],"schema":"https://github.com/citation-style-language/schema/raw/master/csl-citation.json"\}$^[1]^, where D is the flexural stiffness that can be determined by the equation $D=\frac{Et^{3}}{12(1-v^{2})}$ ^[1]^, where E and v are the Yong’s modulus and Poisson’s ratio of the material respectively.

For BOPP, the typical Young’s modulus value of 2.5 GPa in the machine direction ^[2]^ and Poisson’s ratio of 0.3 ^[3]^ are used. For the TPU adhesive film, Young’s modulus and Poisson’s ratio are experimentally determined by tensile testing (Figure S5 and S8). The hinge lengths of both BOPP and TPU hinges are obtained through measurement of straight line hinge length using Tracker ^[4]^, with results as shown in Table S1.

The value of the spring constant of the kinesiology tape elastic element k_KT_, where the elastic element is modelled as a linear spring, can be determined with the equation $k_{\mathrm{KT}}=\frac{E_{\mathrm{KT}}A_{\mathrm{KT}}}{L_{KT,0}}$, where E_KT_, A_KT_ and L_KT,0_ and the Young’s modulus, cross-sectional area, and initial unstretched length of the linear spring respectively. Tensile testing to obtain the Young’s modulus of the kinesiology tape was conducted, with the results as shown in Figure S5. Cross-sectional area of the elastic element is calculated based on the thickness of the kinesiology tape, measured to be around 0.413 mm, and the 10 mm width of tape (width optimized as shown in Figure S9) used for the elastic element, multiplied by two for the two elastic elements used per hinge in the EFRA. As the initial length of the elastic element L_KT,0_ stretched across the hinge cannot be zero and to account for potential delamination and non-zero gap between the plates, the initial length of the elastic element is treated as a fitting factor, determined by minimization of root mean square error with; 1) the experimental results of the contraction ratio with voltage test using bipolar square wave voltage; 2) the experimental results of the blocked force with voltage tests; and 3) the experimental results of the force with displacement tests.

The value of the spring constant k_b2_, similarly is also treated as a fitting factor due to the complexity of experimentally determining the actual value of k_b2_ ^[1]^. The value of k_b2_ is also determined by minimization of the root mean square error with experimental results alongside L_KT,0_. The value of L_KT,0_ and k_b2_ was determined to be 0.85 mm and 7843.37 N/m respectively.

*Relationship between* $\theta$ *and contraction ratio*

To establish the relationship between *θ* and EFRA contraction ratio as described in Section 2.4.2, the inner lumen of the EFRA is simplified into the geometric diagram shown in Figure S6, where the shape of the inner lumen is simplified into a combination of three isosceles triangles and one equilateral triangle as also described in Figure 3a. *θ_j_* is the angle between two plates of an electrohydraulic joint, described by:

$\theta_{j}=180^{\circ}- \theta$ (S1)

, and *θ_h_* is the angle between two plates at the flexible hinge between two electrohydraulic joints, which is expressed by:

$\theta_{h}=60^{\circ}- \theta$ (S2)

The area of each isosceles triangle can be described in the following equation:

$A_{isos}= \frac{1}{2}{l_{3}}^{2}sin(60^{\circ}-\theta)$ (S3)

where *A_isos_* is the area of each isosceles triangle, and *l_3_* is the plate length as shown in Figure 6. To obtain the area of the central equilateral triangle, each side of the equilateral triangle is calculated to be:

$d=2l_{3}sin\left( \frac{60^{\circ}-\theta}{2} \right)$ (S4)

The area of the equilateral triangle may therefore be described in the following equation:

$A_{equi}= \sqrt{3}{l_{3}}^{2}\sin^{2} \left( \frac{60^{\circ}-\theta}{2} \right)$ (S5)

The total area of the inner lumen of the EFRA can therefore be expressed as:

$A_{total}= {l_{3}}^{2}\left[ \frac{3}{2}\sin\left( 60^{\circ}-\theta\right)+\sqrt{3}\sin^{2}\left( \frac{60^{\circ}-\theta}{2} \right) \right]$ (S6)

which is then used in the contraction ratio equation as shown in Equation 1 to create the contraction ratio vs voltage model as shown in Figure 7.

*Modelling of force output*

The force output of the EFRA is modelled as a single representative electrohydraulic joint, which corresponds to the topmost joint that is in contact with the testing bar in the blocked force and force-displacement tests (Figure 4 and Figure 5). In these tests, the constraining bar, the testing bar, as well as the closed-ring structure of the EFRA limit ring closure and restrict motion of the other two joints, and as such the actuator is treated as having approximate symmetrical motion on all three joints. However, the torque contributions of the other two joints are not explicitly included in the force calculation. Force is computed from a single representative joint which is then mapped to the measured inner vertex force output, with the two tips of the representative joint being effectively constrained by the other two joints. The optimized parameters of *k_b2_* and *L_KT,0_* are treated as effective lumped parameters that capture unmodelled compliance and losses in the assembled structure and test interfaces.

For a given bending angle *θ* and actuation voltage, the internal energy of a single electrohydraulic joint is first minimized. The principle of virtual work is then used to obtain the torque output *τ* from the change in minimized energy of the electrohydraulic joint over an infinitesimal angular step. The reaction *F_tip_* at each effectively constrained tip of the electrohydraulic joint as shown in Figure S7 is then calculated based on the torque and the kinematic moment arm. The summation of the forces at both tips is then used to obtain the inner vertex reaction force *F_vertex_*.


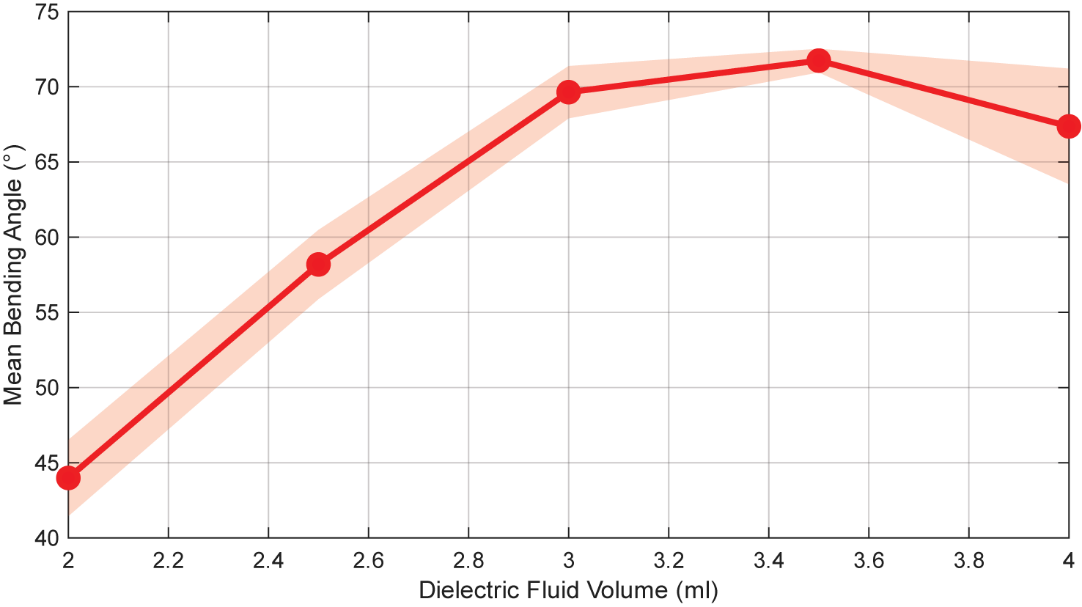


**Figure S1. Dielectric fluid volume optimization for one electrohydraulic joint.** The optimization was conducted with individual electrohydraulic joints without any kinesiology tape or TPU adhesive film. Electrohydraulic joints are actuated for 3 s with bipolar square wave voltage at 8 kV. Data are plotted as mean ± SEM across three samples per dielectric fluid volume, with three trials per sample. Trial data were averaged from 0.3 s after actuation onset to the end of the actuation window to remove initial overshoot.


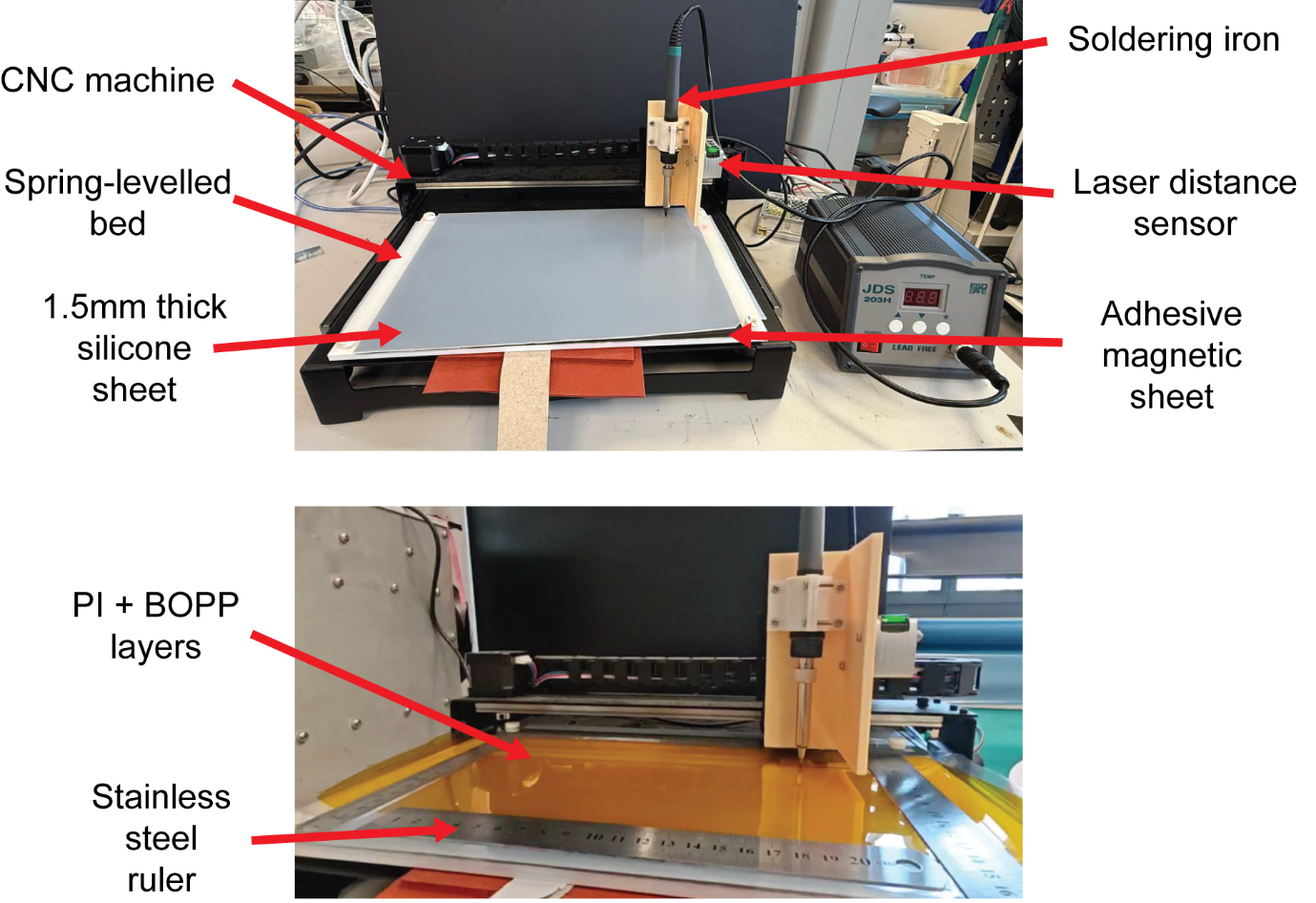


**Figure S2. CNC heat sealer used to heat seal the BOPP pouches.** The heat sealer was modified from a CNC pen plotter (Deep Young Writing Technology) to mount a soldering iron (JDS 203H) with a 3D-printed adapter, as well as to fit a spring-levelled acrylic bed to ensure even distance between the soldering iron and the heat-sealed films at all parts of the bed. A laser distance sensor (BOJKE BGL-235NMZ) is used to ensure accurate levelling of the bed. Stainless steel rulers are used to press out air between the BOPP and PI films before sealing and are also used to hold the films down to the bed by attachment to an adhesive magnetic sheet applied on the acrylic bed. A 1.5 mm thick silicone sheet is also placed underneath the films for even pressure distribution of the soldering iron tip to prevent damage to the BOPP.


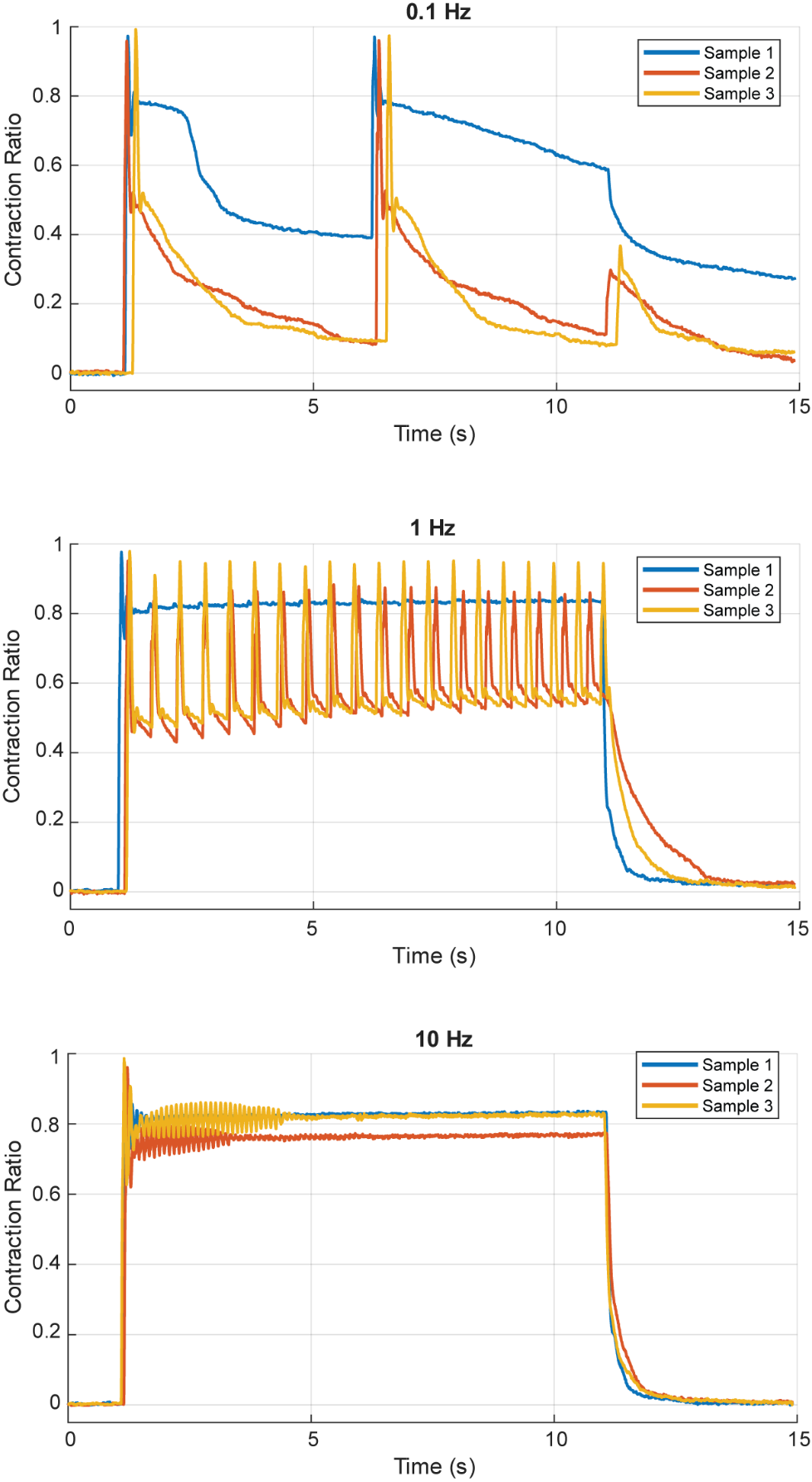


**Figure S3. Contraction ratio vs time plots of a representative trial of each EFRA sample in the frequency-dependent actuation stability tests.** Sample 1 exhibits a slower loss of contraction ratio as opposed to Samples 2 and 3. This variance is likely attributed to inconsistent electrode thickness application on the EFRAs resulting from the manual masking and painting method used to apply the carbon ink electrodes.


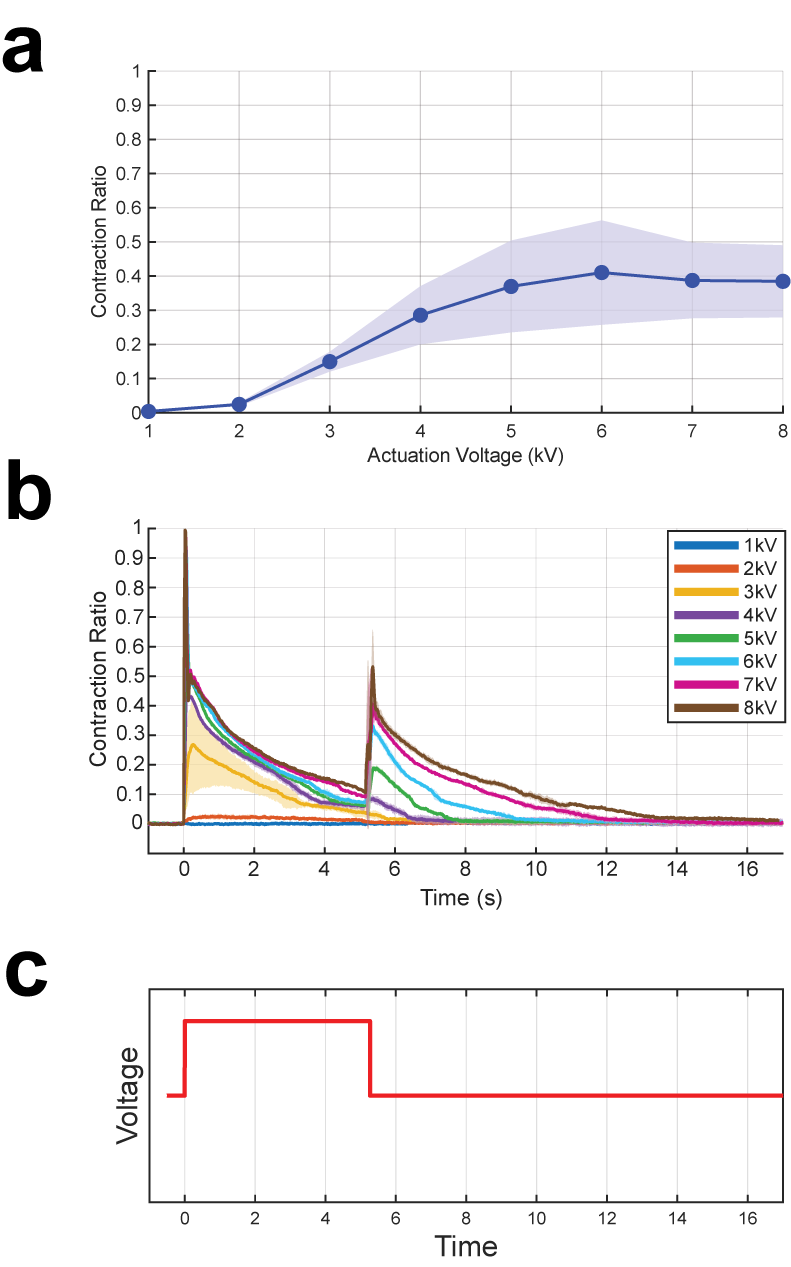


**Figure S4. Radial contraction ratio performance of the EFRA under unipolar square wave voltage.** Results of EFRA under unipolar voltage actuation. Mean contraction ratio vs actuation voltage is plotted as mean ± SEM across three actuator samples (n = 3) with three trials per sample. Contraction ratio vs time is plotted as mean ± SD of one actuator sample (n = 1) across three trials.


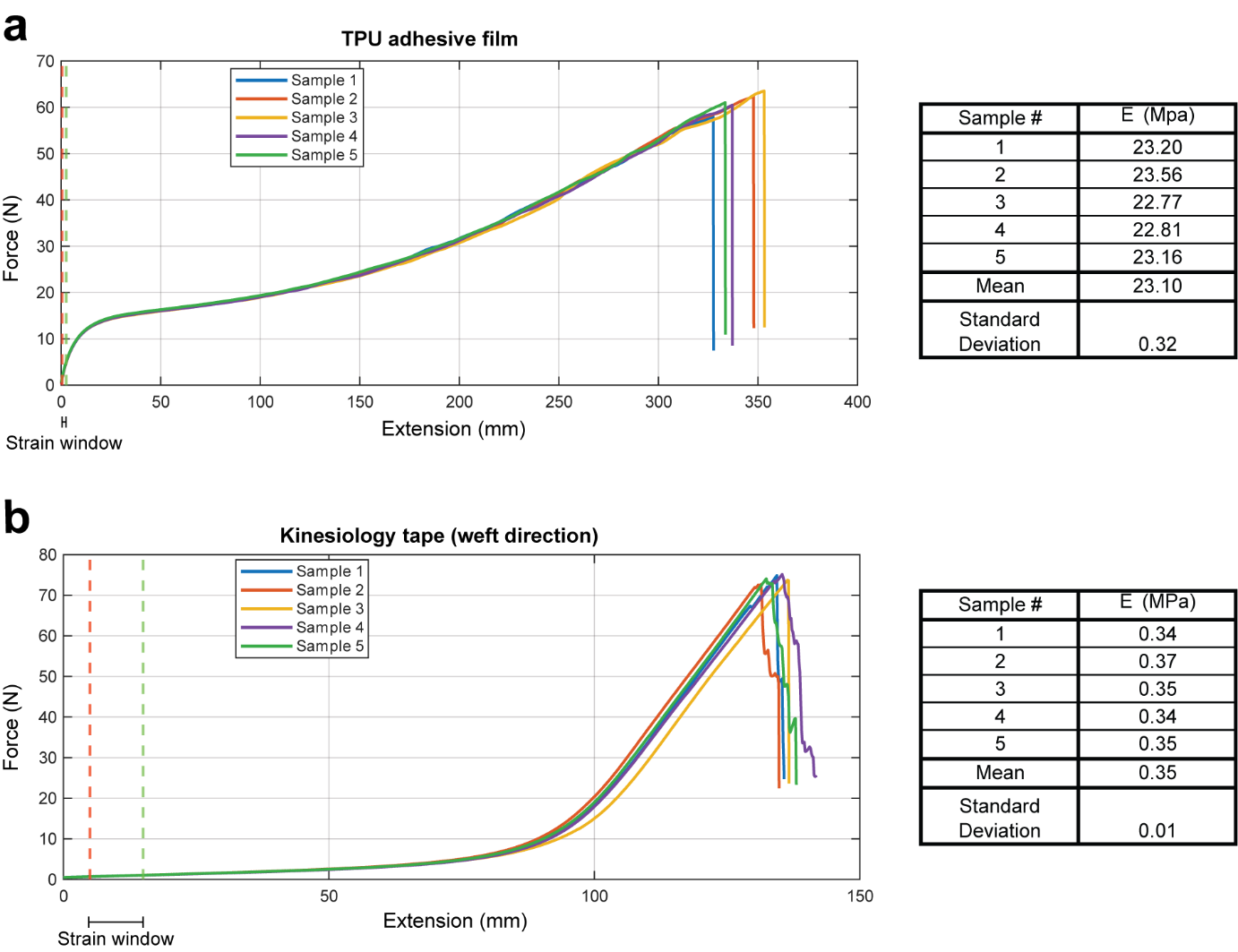


**Figure S5. Force vs extension results collected from tensile testing for the TPU adhesive film and kinesiology tape used in the EFRA to calculate their Young’s modulus.** a) Tensile test for the TPU adhesive film. Five samples of a width of 25 mm were tested, with a gauge length of 50 mm, a prestress of 0.05 % gauge length (0.025 mm), and with a testing speed of 100 mm/min as per testing recommendations from BS EN ISO 527‑1:2019 ^[5]^ and BS EN ISO 527‑3:2018 ^[6]^. b) Tensile test for the kinesiology tape. Kinesiology tape samples are prepared for stretch in the weft direction, as the tape is applied across the hinge of the electrohydraulic joints of the EFRA in the weft direction to allow for stretch. Five samples of a width of 25 mm were tested, with a gauge length of 100 mm, a prestress of 0.5 N, and with a testing speed of 100 mm/min as per recommendations from BS EN ISO 13934-1:2013 ^[7]^.


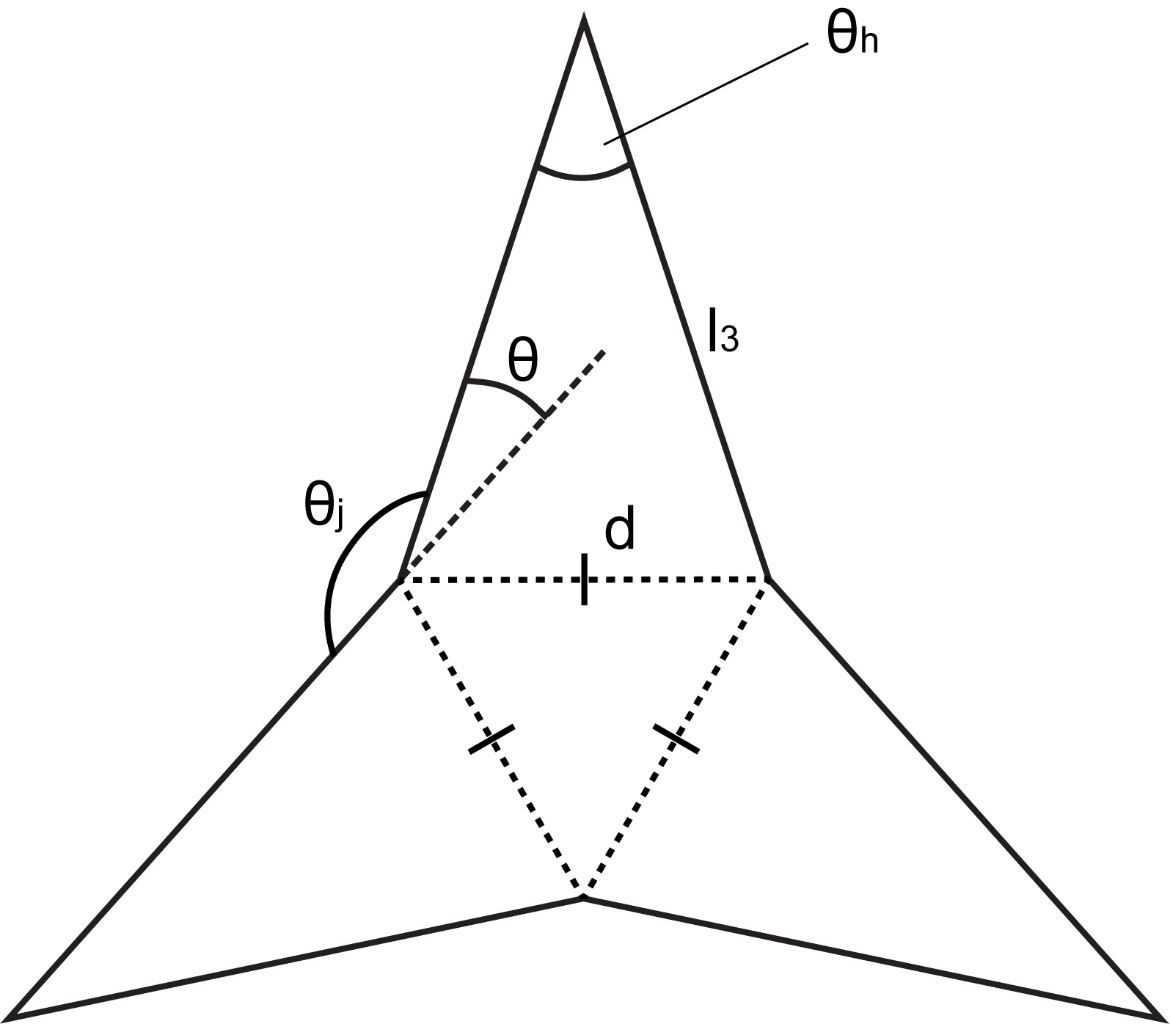


**Figure S6. Simplified geometric diagram of an EFRA.** The outline of the diagram comprises the rigid plates of the EFRA with length *l_3_*_._ *θ_h_* is the angle between the rigid plates at the flexible TPU linkages of the EFRA. *θ_j_* is the angle between the rigid plates of a single electrohydraulic joint. *d* is the length of one side of the equilateral triangle drawn between the three inner vertices of the EFRA.


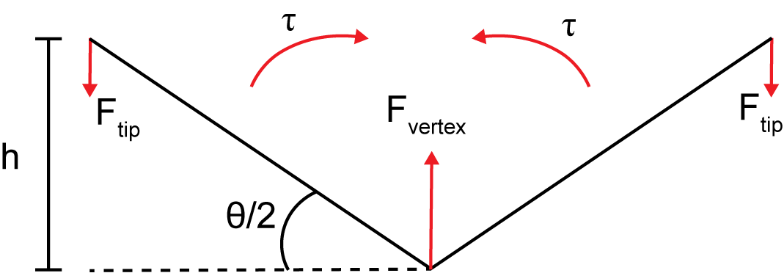


**Figure S7. Force output model diagram.** Torque output *τ* of an electrohydraulic joint is used to calculate downward-directed reaction force *F_tip_* at each effectively constrained tip, which are then summed over both tips to obtain the reaction force F_vertex_ at the vertex of the electrohydraulic joint


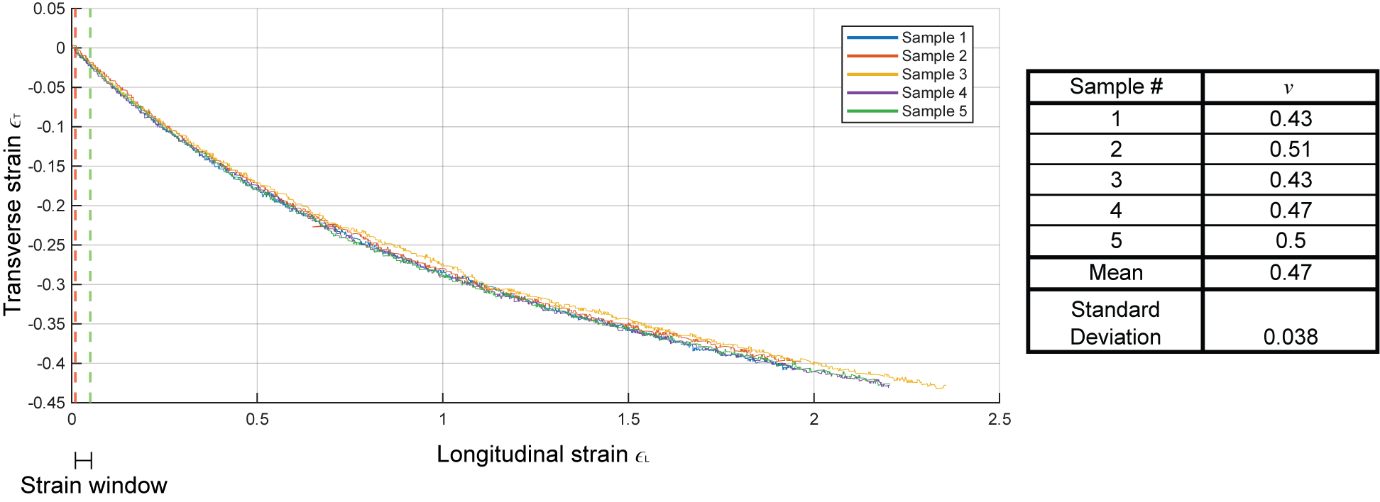


**Figure S8. Transverse strain vs longitudinal strain results from tensile testing for the TPU adhesive film to calculate its Poisson’s ratio.** Results are obtained from the same tensile testing conducted as described in Figure S3(a), but with 240 FPS video taken of the sample, with a transverse line marked in the middle of the gauge length of the sample to allow for tracking of transverse length and therefore transverse strain through motion tracking with Kinovea ^[8]^.


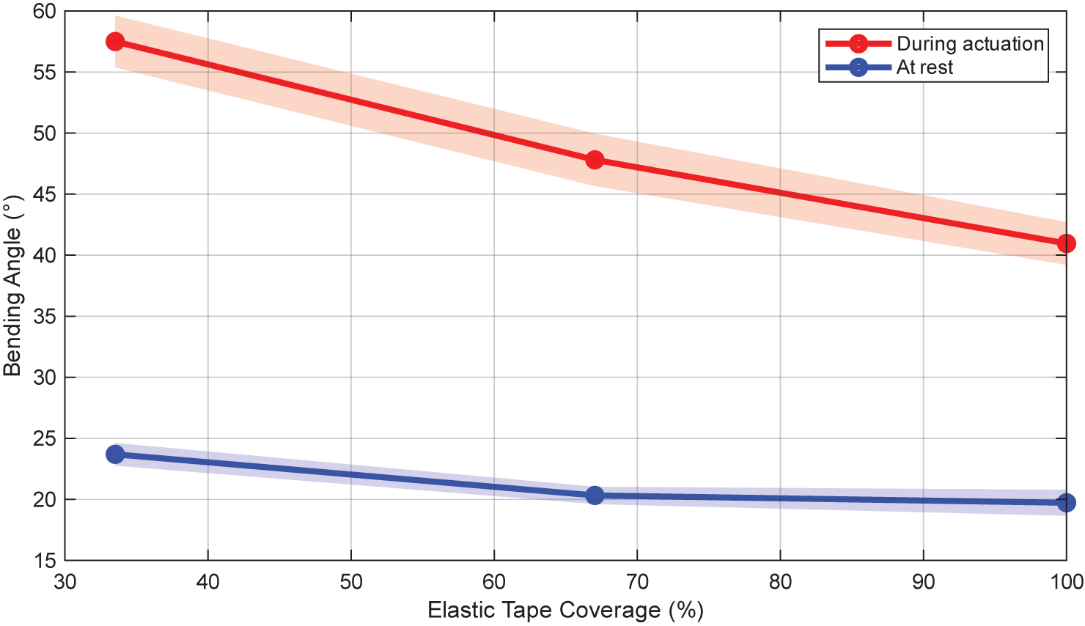


**Figure S9. Optimization of kinesiology tape coverage used for the elastic elements applied across the hinge of the electrohydraulic joints (joint width: 59.67 mm) in the EFRA.** Three different conditions were tested: 1) Two strips of 10 mm width corresponding to 33.5 % coverage, 2) Two strips of 20 mm width corresponding to 67.0 % coverage, and 3) a single strip with full coverage of the joint corresponding to 100 % coverage. Data is plotted as mean ± SEM across three samples, with three trials conducted per sample. Initial bending angle at rest and bending angle during actuation are shown.

| Sample | BOPP hinge length (mm) | TPU hinge length (mm) |
| --- | --- | --- |
| 1 | 0.317 | 1.697 |
| 2 | 0.352 | 1.379 |
| 3 | 0.26 | 1.61 |
| mean | 0.309666667 | 1.562 |

**Table S1. Hinge lengths measured for the BOPP hinge and TPU hinge across 3 actuator samples.** The average hinge length is used for calculation of the spring constant of the rotational springs used to model the BOPP and TPU hinges.

**References**

[1] N. Kellaris, P. Rothemund, Y. Zeng, S. K. Mitchell, G. M. Smith, K. Jayaram, C. Keplinger, *Adv. Sci.* **2021**, *8*, 2100916.

[2] Mitsubishi Polyester Film GmbH, *Comparative data for plastic films*.

[3] H.-Y. Nie, M. J. Walzak, N. S. McIntyre, *Appl. Surf. Sci.* **2006**, *253*, 2320.

[4] D. Brown, *Tracker Video Analysis and Modeling Tool*, **2025**.

[5] British Standards Institution, *Plastics – Determination of tensile properties, Part 1: General principles (ISO 527-1:2019)*, 3rd ed., BSI Standards Limited **2019**.

[6] British Standards Institution, *Plastics – Determination of tensile properties Part 3: Test conditions for films and sheets (ISO 527-3:2018)*, 2nd ed., BSI Standards Limited **2018**.

[7] British Standards Institution, *Textiles — Tensile properties of fabrics, Part 1: Determination of maximum force and elongation at maximum force using the strip method (ISO 13934-1:2013)*, 2nd ed., BSI Standards Limited **2013**.

[8] J. Charmant, *Kinovea*, **2023**.
